# Phosphoproteome dynamics of *Saccharomyces cerevisiae* under heat shock and cold stress

**DOI:** 10.15252/msb.20156170

**Published:** 2015-06-03

**Authors:** Evgeny Kanshin, Peter Kubiniok, Yogitha Thattikota, Damien D'Amours, Pierre Thibault

**Affiliations:** 1 Institute for Research in Immunology and Cancer, Université de MontréalMontréal, QC, Canada; 2 Department of Chemistry, Université de MontréalMontréal, QC, Canada; 3 Department of Pathology and Cell Biology, Université de MontréalMontréal, QC, Canada; 4 Department of Biochemistry, Université de MontréalMontréal, QC, Canada

**Keywords:** cold stress, dynamics, heat shock, phosphoproteomics, signaling

## Abstract

The ability of cells and organisms to survive and function through changes in temperature evolved from their specific adaptations to nonoptimal growth conditions. Responses to elevated temperatures have been studied in yeast and other model organisms using transcriptome profiling and provided valuable biological insights on molecular mechanisms involved in stress tolerance and adaptation to adverse environment. In contrast, little is known about rapid signaling events associated with changes in temperature. To gain a better understanding of global changes in protein phosphorylation in response to heat and cold, we developed a high temporal resolution phosphoproteomics protocol to study cell signaling in *Saccharomyces cerevisiae*. The method allowed for quantitative analysis of phosphodynamics on 2,777 phosphosites from 1,228 proteins. The correlation of kinetic profiles between kinases and their substrates provided a predictive tool to identify new putative substrates for kinases such as Cdc28 and PKA. Cell cycle analyses revealed that the increased phosphorylation of Cdc28 at its inhibitory site Y19 during heat shock is an adaptive response that delays cell cycle progression under stress conditions. The cellular responses to heat and cold were associated with extensive changes in phosphorylation on proteins implicated in transcription, protein folding and degradation, cell cycle regulation and morphogenesis.

## Introduction

Single cell organisms such as yeast are regularly exposed to environmental changes that required the development of adaptation mechanisms for survival in suboptimal growth conditions. Variation in temperature is a common stress experienced by yeast cells, a condition for which evolutionary changes favored organisms that developed appropriate responses to heat shock and cold stress. For example, *S. cerevisiae* have evolved protective transcriptional programs in response to elevated temperature (> 37°C) that result in physiological changes affecting carbohydrate flux, cytoskeleton dynamics and protein folding (Morano *et al*, 2012; Verghese *et al*, 2012). The molecular basis of the response to different stresses including heat shock has been well documented in *S. cerevisiae*, and genome-wide transcriptional profiling studies identified important regulators of environmental stress response (ESR) (Gasch *et al*, 2000; Gasch & Werner-Washburne, 2002). A large proportion of induced ESR genes target different cellular processes, including carbohydrate metabolism, protein folding and degradation, autophagy, cytoskeletal reorganization and DNA damage repair. In contrast, ESR genes with repressed expression are involved in protein synthesis, ribosome synthesis and processing, RNA polymerase-dependent transcription and protein translation.

Short-term exposure to moderate heat shock has been reported to induce a complex cellular response culminating in a transient cell cycle arrest at the G1/S transition in both yeast and mammalian cells (Kuhl & Rensing, 2000). This response appears to be associated with the activity threshold of cyclin-dependent kinases (Kuhl & Rensing, 2000), and overexpression of G1 cyclins in yeast has been shown to overcome the transient arrest induced by heat shock (Rowley *et al*, 1993). Moreover, Ste20, Cla4 and Skm1, all members of the p21-activated kinase (PAK) family, are also activated upon changes in temperature and play important roles in the regulation of osmosensing, filamentous growth and septin ring assembly (Holly & Blumer, 1999; Raitt *et al*, 2000b; Gancedo, 2001; Versele & Thorner, 2004). Interestingly, decreased levels of the molecular chaperone Hsp90 or its kinase-specific chaperone Cdc37 are known to affect Cdc28 and Cla4 stability (Farrell & Morgan, 2000), a condition that favors a subset of cells to switch to a filamentous form in response to hostile environment (Hsieh *et al*, 2013).

While a large body of the literature exists on the molecular basis of heat shock in yeast, mechanisms associated with the response to cold stress are still poorly understood (Aguilera *et al*, 2007). Experimental evidences suggest that changes in membrane fluidity function as a cold sensor to trigger subsequent signal transduction (Los & Murata, 2004). Exposure to low temperature induces the accumulation of trehalose, glycerol and heat-shock proteins to protect yeast cells against freeze injury (Schade *et al*, 2004). Both heat shock and cold stress appear to be regulated by classical stress pathways involving the high-osmolarity glycerol mitogen-activated protein kinase Hog1, and the transcriptional factors Msn2 and Msn4 (Panadero *et al*, 2006). Aside from the classical stress pathways in which Hog1 is playing a central role, little is known about the signaling events resulting from changes in temperature.

To gain further insights on the dynamic changes in protein phosphorylation associated with heat shock and cold stress, we performed a large-scale phosphoproteomics study on *S. cerevisiae*. Yeast represents one of the most intensively studied eukaryotic cell model organism with a relatively simple genome consisting of ~6,500 genes. The introduction of sensitive mass spectrometry (MS) instrumentation and affinity media for phosphopeptide enrichment has paved the way to comprehensive phosphoproteome analysis that significantly expanded the repertoire of protein phosphorylation identified in yeast and other model organisms. Several large-scale quantitative phosphoproteomics studies conducted on yeast cells have already described changes in protein phosphorylation in response to different stimuli including pheromone (Gruhler *et al*, 2005), DNA damage (Smolka *et al*, 2007), fatty acid and peroxisome induction (Saleem *et al*, 2010) and kinase inhibition (Bodenmiller *et al*, 2010). Other large-scale studies using stable isotope labeling in cell culture (SILAC) highlighted the regulation of protein phosphorylation in yeast during the cell cycle (Holt *et al*, 2009), and as a function of ploidy (de Godoy *et al*, 2008). While MS-based phosphoproteomics enables the profiling of thousands of phosphopeptides from microgram amounts of sample, the identification of the phosphosites that are specific to the stimulus remains a challenge, due to the prevalence of promiscuous phosphorylation events arising from random encounters of kinases with abundant neighboring proteins (Levy *et al*, 2012). The identification of differentially regulated phosphoproteins by conventional data analysis approaches typically relies on fold change (FC) in phosphopeptide abundances taken at different time points shortly after the onset of the stimulus. However, dynamic changes in protein phosphorylation are difficult to establish based on the FC measurements obtained from a limited number of time points. We reasoned that the acquisition of high-resolution kinetic profiles could provide valuable information on phosphoproteome dynamics to further understand how temporal changes in kinase and phosphatase activities are reflected on downstream substrates in response to environmental stress.

Accordingly, we performed quantitative phosphoproteomics analyses using SILAC (Ong & Mann, 2006) and profiled changes in protein phosphorylation for the first 28 min upon temperature stress with a temporal resolution of 2 min. The ability to determine kinetic changes in protein phosphorylation with high resolution enabled the identification of 5,554 high-quality profiles (≥ 10 measurements out of 15), including dynamic phosphorylation profiles of low amplitude that typically elude conventional phosphoproteomics approaches based on FC alone. We identified 391 phosphosites that showed distinct changes in phosphorylation upon heat shock or/and cold stress. Detailed clustering analyses of dynamic profiles distinguished different types of patterns such as monotonous and adaptation-like responses. Also, we observed that kinases shared similar kinetic profiles with known substrates, a correlation that facilitated the identification of other putative substrates. Phosphosites exhibited notable differences of dynamic patterns in response to cold and heat, underlining the complexity of signaling rewiring essential for cell adaptation and survival. The ability to profile phosphoproteome dynamics and identify stimulus-specific phosphosites may provide additional insights on the regulation of protein phosphorylation in a wide variety of cellular processes.

## Results

### High temporal resolution of the *S. cerevisiae* phosphoproteome dynamics in response to heat and cold

**Figure 1:**
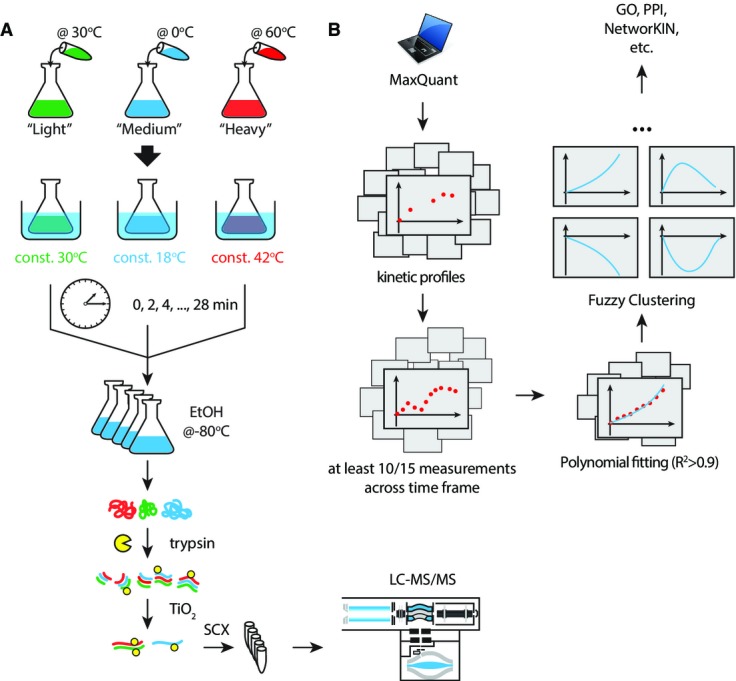
Experimental design and data analysis workflow

We designed an experimental workflow to measure phosphoproteome dynamics with 2-min temporal resolution and monitor changes associated with heat shock and cold stress in *S. cerevisiae*. Briefly, cells were grown at 30°C in media containing light (^0^Lys, ^0^Arg), medium (^4^Lys, ^6^Arg) or heavy (^8^Lys, ^10^Arg) isotopic forms of lysine and arginine. Cell cultures were mixed with either cold or hot fresh media and maintained in a thermostated water bath to induce a rapid shift in temperature to 18°C (cold stress), 42°C (heat shock) or 30°C (control) (Fig1A). Aliquots from each culture were collected every 2 min and pooled together in cold (−80°C) ethanol to prevent protein degradation and changes in phosphorylation. After centrifugation, cells were lysed by bead beating in urea buffer, and proteins were digested into peptides with trypsin. Phosphopeptides were enriched on TiO_2_ affinity resin and fractionated offline into six SCX salt fractions prior to LC-MS/MS analysis. Phosphopeptide identification and quantification was performed using MaxQuant (Cox & Mann, 2008; Cox *et al*, 2011b), and further analyses were achieved using R (http://www.r-project.org/), Perl (www.perl.org) and bioinformatics tools such as KinomeXplorer (Horn *et al*, 2014) and STRING (Franceschini *et al*, 2013) (Fig1B and Materials and Methods). We identified a total of 9,357 unique phosphosites on 2,344 proteins, of which ~70% were localized with high confidence (6,553 phosphosites). The abundance of phosphopeptides was profiled across time, and only those quantified in at least 10 out of 15 time points were retained for further analysis. Accordingly, we identified 5,554 high-quality profiles corresponding to 2,777 phosphosites (1,228 proteins) (Supplementary Fig S1 and Supplementary Table S1). A pairwise comparison of phosphopeptide abundance across time points is provided in Supplementary Fig S2.

Temperature shifts are initiated by adding either hot (60°C) or cold (0°C) media to cell cultures. The temperature of cultures is maintained in thermostated water bathes throughout the duration of the experiments. Samples are collected every 2 min by mixing yeast cultures in cold ethanol to immediately preserve protein phosphorylation. Yeast cells are centrifuged and lysed under denaturing conditions, and extracted proteins are digested with trypsin. Phosphopeptides are purified on TiO_2_ beads and fractionated offline on SCX spin columns prior to LC-MS/MS analyses.

Phosphopeptide identification and quantitation is performed using MaxQuant, and only kinetic profiles containing at least 10 time measurements were selected for further analysis. Polynomial fitting was used to define regulated phosphosites. Sites with *R*^2^ > 0.9 were selected and used for fuzzy c-means clustering and subsequent analyses.

**Figure 2:**
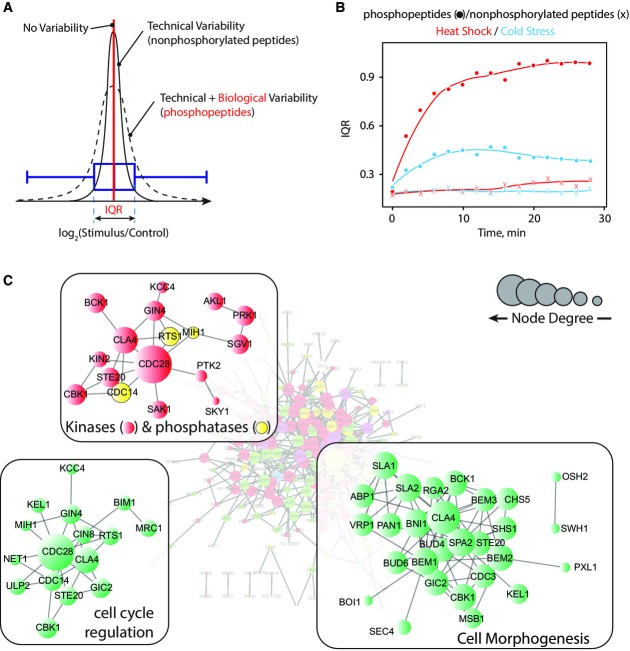
Proteome-wide effects of temperature

To determine the overall changes in phosphoproteome in response to heat shock or cold stress, we used fold change (FC) ratio of peptide abundance between conditions as a proxy for biological variability induced by the stimulus (Fig2A). Global effects of the treatment (heat or cold) on the phosphoproteome was evaluated from the width of the log_2_ FC distribution of all phosphopeptides for a given time point. The width of this distribution is represented as the interquartile range (IQR). If the treatment produces global changes in phosphorylation, this will be reflected by a widening of the FC distribution. We plotted the interquartile ranges (IQR) for log_2_(FC) distributions of phosphopeptides to monitor the global changes of the phosphoproteome upon heat and cold stresses (Fig2B). The IQR values of nonphosphorylated peptides retained on TiO_2_ in a nonspecific manner are also shown for comparison. At the onset (T = 0 min), no significant differences were observed in IQR values for all conditions, and coefficient of variation for abundance measurements was within ± 0.29 for all quantified peptides. A progressive increase in FC ratio of phosphopeptides was observed during the first 10 min upon heat shock and cold stress while the abundance of nonphosphorylated peptides remained relatively constant over the entire time period of the experiment. It is noteworthy that changes in phosphorylation were markedly different between stimuli, suggesting that exposure to elevated temperature has more pronounced effects on the phosphoproteome of *S. cerevisiae* with FC ratios typically four times higher on average for heat shock compared to cold stress. Protein phosphorylation reached a plateau around 20 min upon stimulation, possibly reflecting a gradual adaptation of yeast cells to the environmental condition.

Width of FC distribution is used to define biological variability associated with a particular stimulus. No significant changes in the abundance of nonphosphorylated peptides were observed during the experiment, and the corresponding coefficient of variation was used to determine technical variability (sample processing and LC-MS/MS analysis). The FC distribution of phosphopeptides corresponds to the sum of technical variability and stimulus-associated changes.

Phosphopeptides exhibited a progressive increase in IQR with time, reflecting the global effects of temperature on the phosphoproteome. Notably, heat shock produced more pronounced effects compared to cold stress. The distribution of FC for nonphosphorylated peptides remained largely unaffected.

Proteins that displayed changes in phosphorylation upon temperature shifts were used to build interaction network using the STRING database (http://string-db.org/), and selecting high stringency (score > 0.7) interactions inferred from experimental data. Node degree is the number of interacting partners in the network. These analyses revealed that responses to heat shock and cold stress are complex and involved the activation of many kinases and phosphatases that affect downstream substrates with wide range of cellular functions, including cell cycle regulation and cell morphogenesis.

In order to define biologically regulated phosphosites in the dataset, we first performed fitting of all kinetic profiles with a polynomial model, selecting only those with *R*^2^ > 0.9 (Fig1B and Supplementary Fig S3). This analysis does not depend on the absolute ratios of abundance measurements or fold change (FC); rather, it is based on the shape and low variance (i.e. low RSD values of SILAC measurements) of kinetic profiles. Profiles with high RSD do not generally provide meaningful *R*^2^ values and are not considered for subsequent analyses. It is noteworthy that FC values do not reflect actual stoichiometry of phosphorylation. For example, a FC value of 10 might indicate a change in phosphorylation from 1 to 10%, whereas a FC value of two could correspond to full protein phosphorylation (from 50 to 100%). Since biological relevance of phosphosite cannot be based on FC measurements alone, profiles with low amplitude (small FC) could be as important as profiles with high amplitude (high FC). In this study, we assign significance based on the shape of kinetic profiles rather than absolute values of the corresponding FC values. Therefore, this approach enabled the selection of profiles with low amplitude that could elude conventional quantitative phosphoproteomic methods based on FC alone. We performed fuzzy clustering of these profiles and selected only profiles with cluster membership value higher than 0.7 for subsequent analyses. We identified 388 phosphosites from 271 proteins that showed distinct changes in phosphorylation (increase or decrease) with time for at least one stimulus (Supplementary Table S2). For convenience, we hereafter defined these phosphosites as “dynamic” to distinguish them from remaining “static” phosphosites. Dynamic sites showed significant changes in phosphorylation where max |log_2_ FC| measurements were typically two-fold higher than static sites (Supplementary Fig S4). To account for the possibility that changes in protein abundance might affect the interpretation of regulated phosphosites, we performed experiments on flow through fractions containing nonphosphorylated peptides from TiO_2_ enrichment experiments. Samples were desalted and fractionated on a PGC column into 40 fractions prior to LC-MS/MS analyses. These experiments enabled the quantitation of ~85% of all dynamic phosphoproteins reported in Supplementary Table S2. We observed that only two proteins showed more than 2 fold changes in abundance over the entire time period of the experiment. Accordingly, Hsp42 (log_2_FC: 2.06) and Zeo1 (log_2_FC: 1.33) were excluded from the list of dynamic phosphoproteins. The list of quantified proteins and their corresponding changes in protein abundance is reported in Supplementary Table S3.

To find the relationships among dynamic phosphoproteins that contained at least one dynamic phosphosite, we integrated our identification into protein–protein interaction (PPI) network (Fig2C) and performed GO enrichment analysis (Huang da *et al*, 2009b) (Supplementary Fig S5 and Supplementary Table S4). Cell response to changes in temperature appears to be complex and comprised a network of interacting proteins involved in cytokinesis, cell morphogenesis and cell cycle regulation. Changes in phosphorylation are consistent with a kinase–phosphatase network associated with the response to temperature stress, and we observed that about 15% (20/129) of all kinases and 7% (2/30) of all phosphatases also comprised dynamic sites (Fig2C and Supplementary Table S6). Several of these kinases including Rck2 and Hog1 are also regulated upon environmental stress including osmotic shock (Bilsland-Marchesan *et al*, 2000).

### Temporal phosphorylation profiles identify potential kinase substrates in response to heat shock and cold stress

**Figure 3:**
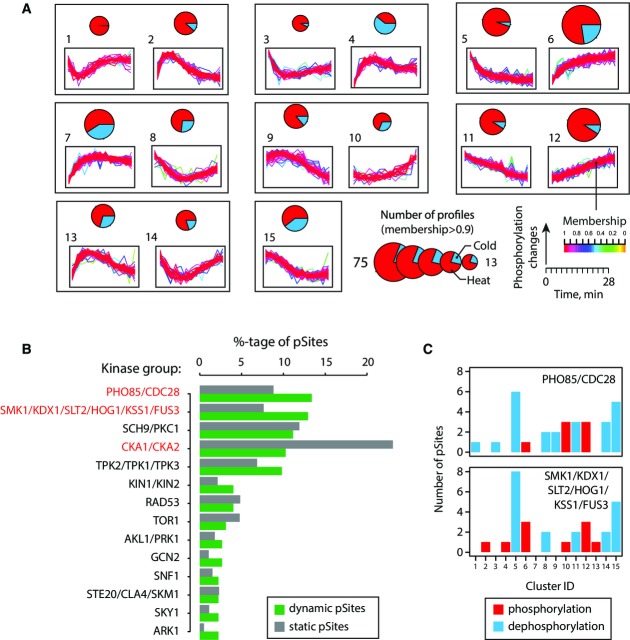
Types of dynamic behavior and kinases involved in response to heat and cold stresses

To gain further insights on kinetic profiles shared among dynamic phosphosites, we analyzed our dataset using fuzzy c-means clustering and selected only clusters with membership > 0.7 (Figs1B and 3A). These analyses enabled the separation of 388 dynamic phosphosites from 271 proteins into 15 different clusters with unimodal distributions showing monotonous (clusters 5, 6 and 9–12) and adaptation-like (clusters 1–4, 7, 8 and 13, 14) profiles (Supplementary Table S2). With the exception of clusters 9–12, the temporal variation in FC (increase or decrease) of several profiles reached a maximum value within 20 min upon temperature shift. Several profiles showed changes in phosphorylation that returned to their original status within 30 min post-stimulation. For example, this is the case for the kinase Hog1, and the transcriptional factors Msn2 and Msn4 that were previously reported to be regulated by changes in temperature (Panadero *et al*, 2006). It is noteworthy that phosphosites from the same protein can exhibit different profiles and that individual phosphosite often showed different behaviors in response to heat and cold. For example, we identified five dynamic sites on trehalose synthase complex regulatory subunit, Tsl1 (S53, S73/S77, S147 and S161), of which two sites (S147 and S161) showed opposite trends upon heat shock and cold stress. Tsl1 is a regulatory subunit of the trehalose synthesis protein (TSP) complex that catalyses the production of trehalose from glucose-6-phosphate and UDP-glucose, an activity mediated by heat shock (Ferreira *et al*, 1996). Changes in the phosphorylation status of Tsl1 may regulate its association with the TSP complex under stress condition (Supplementary Fig S6).

Fuzzy c-means clustering of regulated phosphosites with different kinetic profiles. Pie charts represent the distribution of profiles for heat (red) and cold (blue), while circle sizes correspond to the number of high membership profiles. Clusters are ordered according to similarities in their dynamic behavior for either up- or downregulation.

Putative kinases associated with dynamic and static phosphosites based on KinomeXplorer predictions. Substrates for each kinase group are represented by bar plots. A relative enrichment of cyclin-dependent and MAP kinase substrates is observed among dynamic phosphosites, while a higher proportion of casein kinase 1/2 substrates is found for static phosphosites.

Distribution of kinetic patterns among dynamic phosphosites associated with Cdk and MAPK kinase groups. Putative Cdk and MAPK substrates were found to be represented mostly in cluster 5 which correspond to a sustained dephosphorylation.

Several kinases and phosphatases were dynamically affected by changes in temperature and exhibited different kinetic profiles. For example, several sites of important kinases involved in stress response (cluster 2: Hog1 Y176), cell cycle regulation (cluster 6: Cdc28 Y19), nutrient sensing (cluster 8: Gcn2 S577) or cytokinesis (cluster 9: Cdc3 S503) were grouped in different clusters, suggesting distinct temporal regulatory activities. Based on this observation, we surmised that dynamic behaviors of kinase and phosphatase groups could be correlated based on their temporal profiles and that substrates may exhibit similar trends. To test this proposal, we first associated dynamic and static phosphosites with assigned kinase groups based on the predictions made by KinomeXplorer (Horn *et al*, 2014), a platform that integrates an improved NetworKIN algorithm for modeling kinase signaling networks by combining linear motif analysis with protein–protein interactions. We found that ~40% of all dynamic phosphosites are associated with three main kinase groups represented by cyclin-dependent, mitogen-activated and AGC family kinases and that a higher proportion of dynamic sites compared to static sites is observed for the first two groups (Fig3B). These kinases are known to play multiple roles in the regulation of cellular response to environmental conditions and integration of stress signals. The only group of kinases significantly underrepresented among dynamic phosphosites is casein kinases (Ycks). The relative proportion of enrichment or depletion of dynamic sites from other kinase groups is more difficult to establish as the numbers of affected phosphosites are small (< 10 sites).

Next, we regrouped dynamic phosphosites associated with specific kinases with their corresponding clusters to determine whether phosphosites that were found to be phosphorylated by the same putative kinases were grouped in the same cluster. These analyses revealed that dynamic phosphosites attributed to specific kinases could be represented by different clusters (Supplementary Fig S7). This observation is not entirely unexpected since the phosphorylation status of any given site may be affected by several factors including the activity of other kinases and phosphatases, the subcellular location of protein substrates and the creation of binding sites modulating the interactions with proteins containing phosphobinding domains. While temporal profiles associated with specific kinases appeared to be complex, we also noted that several dynamic sites/clusters were more frequently represented in selected kinase groups (Fig3C). For example, cluster 6 contains the cyclin-dependent kinase Cdc28, a major component of the cell cycle control system. The increase in phosphorylation of Cdc28 at the inhibitory site Y19 (Keaton *et al*, 2007) is also correlated with a corresponding dephosphorylation of known Cdc28 substrates found in cluster 5 such as phosphatase Cdc14 (S429), transcription factor activator of CUP1 expression Ace2 (S253) and MAP kinase Ste20 (S547, S562) (Holt *et al*, 2009). Similarly, the increase in activity of Hog1 (Y176) is also correlated with an increase in phosphorylation of known substrates including the transcription factor Sko1 (T215) and Rck2 kinase (S46/S50) that were grouped under the same temporal profiles (cluster 2).

**Figure 4:**
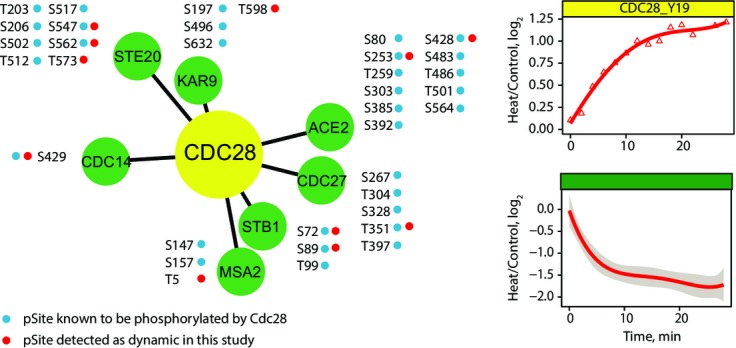
Cdc28 substrates display similar dynamic behavior An increased phosphorylation of Cdc28 at its inhibitory site Y19 was paralleled by a corresponding dephosphorylation on putative protein substrates. STRING was used to obtain high-confidence Cdc28 interactors among proteins with dynamic phosphosites, and known sites regulated by Cdc28 were inferred using PhosphoGRID. A total of eight dynamic phosphosites (red dots) with similar kinetic profiles were identified in our dataset (shaded area across averaged profile represents 95% confidence interval).

The observation that changes in phosphorylation profiles of kinases are associated with those of known substrates suggests that dynamic patterns could be used to identify putative kinase substrates. To explore this possibility, we focused on profiles related with changes in Cdc28 activity upon heat shock. First, we used STRING to identify dynamic phosphoproteins known to interact with Cdc28, based on interactions inferred from experimental data and assigned with the highest confidence (> 0.9) (Franceschini *et al*, 2013). Next, for these proteins we obtained information on sites phosphorylated by Cdc28 based on *in vivo* phosphorylation data from the PhosphoGrid database (Stark *et al*, 2010; Sadowski *et al*, 2013). These analyses identified 32 target phosphosites, of which nine phosphosites were detected in our study as dynamic, and displayed similar patterns of dephosphorylation associated mainly with cluster 5 (Fig4 and Supplementary Fig S8). It is noteworthy that protein tyrosine phosphatase Mih1 (S82), which targets Y19 residue on Cdc28, showed a similar dephosphorylation profile. In addition to Cdc14 (S429), Ace2 (S253, S428), Ste20 (S547, S562), Cdc27 (T351) and Stb1 (S72, S89) that were previously known as Cdc28 substrates, these analyses uncovered new putative sites on Kar9 (T598) and Msa2 (T5) along with a novel site on Ste20 (T573). To confirm putative Cdc28 substrates, we also performed large-scale phosphoproteomics analyses on a yeast strain where the WT Cdc28 was replaced with an analog-sensitive mutant Cdc28-*as1* expressed in the same strain background (Holt *et al*, 2009). These experiments were carried out on six biological replicates with reverse SILAC labeling and confirmed the dephosphorylation of 2 out of 3 putative substrates upon Cdc28-*as1* inhibition with 1NM-PP1: Msa2 T5 (log_2_FC: −4.68, −log_10_(*P*-value): 2.48) and Ste20 T573 (log_2_FC: −2.38, −log_10_(*P*-value): 3.06). Unfortunately, the Kar9 T598 was not detected in these experiments due to its low abundance. The complete list of quantified phosphosites obtained from the inhibition of the analog-sensitive mutant of Cdc28 is presented in Supplementary Table S6.

To gain further insights on the inference of kinase–substrate relationships based on dynamic profiles, motif analyses and protein–protein interactions, we also compared our dynamic phosphosites with those identified in a separate large-scale phosphoproteomics study performed on 116 gene deletion mutants of the nonessential kinases or phosphatases, and 8 analog-sensitive kinase strains of some essential kinases (Supplementary Table S7) (Bodenmiller *et al*, 2010). These analyses revealed that 43% of our dynamic phosphosites from monophosphorylated peptides (134 phosphosites) were also found to be regulated in the system-wide perturbation of kinase and phosphatase study by Bodenmiller *et al* (Supplementary Fig S9A) (Bodenmiller *et al*, 2010). While ~two-third of these sites were regulated by multiple kinases, we found that 42 phosphosites were associated with a single kinase (Supplementary Fig S9B). Closer examination of regulated phosphosites common to both large-scale phosphoproteomics studies indicated an enrichment of some kinases in specific clusters (Supplementary Fig S9C). For example, putative Cdc28 substrates were identified in cluster 5 consistent with that observed previously using KinomeXplorer (Fig3).

**Figure 5:**
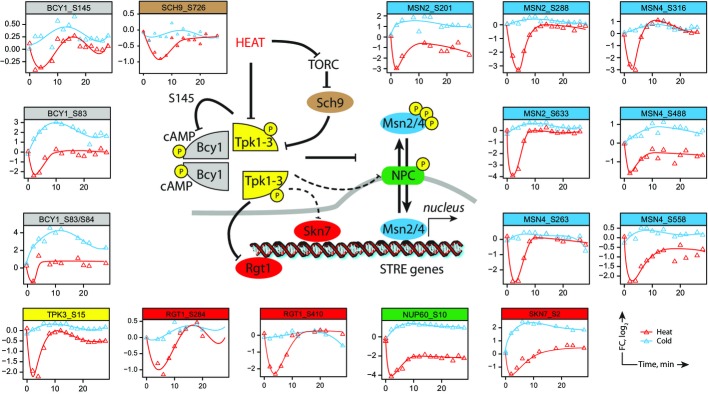
Dynamic profiles reveal functional association between kinases and putative substrates Schematic representation of cell signaling pathway associated with PKA and downstream substrates. Phosphorylation profiles are shown for each substrate site. The kinetic profiles of PKA (Tpk3 and Bcy1) are superimposable with those of known protein targets Msn2/4 and Rgt1. The correlation between temporal profiles was used to identify putative targets of PKA such as nucleoporin Nup60 and the transcription factor Skn7.

Our dataset also contained several phosphosites that showed rapid dephosphorylation upon heat shock followed by a recovery to basal level within 10 min (Fig5). Among these are known examples of transient dephosphorylation of Msn2 and Msn4, two transcription factors that are activated upon cellular stress conditions and rapidly undergo nuclear translocation (Jacquet *et al*, 2003). The activation of Msn2/4 is associated with the rapid but transient dephosphorylation of several sites within the nuclear localization signal (NLS) (De Wever *et al*, 2005). Previous studies indicated that NLS of Msn2 is a direct target of cAMP-dependent protein kinase (PKA) that plays important roles in nutrient sensing (Gorner *et al*, 2002). Under heat shock, we identified three sites on Msn2 (S201, S288 and S633) and four sites on Msn4 (S263, S316, S488 and S558), all located within PKA recognition motif.

In this study, we observed the dephosphorylation of both PKA catalytic subunit Tpk3 (S15) and the regulatory subunit Bcy1 (S83, S84, S145) (Fig5). The phosphorylation of Bcy1 at S145 by Tpk1-3 is required to establish a negative feedback loop in response to changes in nutrient concentrations (Budhwar *et al*, 2010). Tpk3, Bcy1 and Msn2/4 all showed similar temporal phosphorylation profiles, thus providing a dynamic link between these proteins. Upon heat shock, PKA is transiently deactivated which leads to rapid dephosphorylation of Msn2/4 favoring its nuclear translocation. The comparison of kinetic profiles similar to those of Msn2/4, Tpk3 and Bcy1 (cluster 1) also identified other putative PKA substrates including the nuclear pore protein Nup60 (S10), the transcription factors Skn7 (S2) and Rgt1 (S284, S410), the inhibitor of glycogen debranching Igd1 (S83) and a protein of unknown function associated with cell cycle regulation YPL247C (S12) (Fig5). Rgt1 regulates the expression of HXT genes encoding glucose transporters and is phosphorylated *in vitro* by all three isoforms of PKA at serine residues located within consensus sites (Kim & Johnston, 2006). Igd1 is also involved in glucose metabolism and was reported to act as an inhibitor of Gdb1p by enhancing the ability of yeast cells to store glucose as glycogen. The phosphorylation of Igd1 could regulate its interactions with Gdb1 (Walkey *et al*, 2011). The dephosphorylation of the nuclear response regulator and transcription factor Skn7 (S2) through the transient deactivation of Tpk3 might be required for the optimal induction of heat-shock genes in response to oxidative stress (Raitt *et al*, 2000a).

We also observed the dephosphorylation of an upstream regulator of PKA activity—the yeast ortholog of mammalian PKB/Akt and target of rapamycin complex 1 (TORC1) effector, Sch9 protein kinase. Residue S726, which is located within the AGC-kinase domain, is a substrate of TORC1 and was dephosphorylated upon heat shock. Phosphorylation of this residue is lost upon rapamycin treatment and carbon or nitrogen starvation and is transiently reduced following the application of osmotic, oxidative or thermal stress (Roelants *et al*, 2004; Urban *et al*, 2007). Consistent with this result, we also identified phosphorylation sites on Tco89 (S84) and Kog1 (S1045), two components of TORC1 that were dephosphorylated upon heat shock. It is noteworthy that PKA activity is modulated by changes in cAMP concentrations, and we observed an increase in phosphorylation of residue T617 from adenylate cyclase Cyr1 upon heat shock. Residue T617 is located next to the Ras-associating domain of Cyr1, and its kinetic profile showed an opposite trend to that observed for the catalytic subunit Tpk3 (S15). Taken together, our results suggest that the combination of protein–protein interactions and phosphorylation dynamics provides a valuable approach to identify putative kinase substrates and prioritize large-scale phosphoproteomics data for subsequent experiments.

### Differential regulation of signaling pathways upon heat shock and cold stress

**Figure 6:**
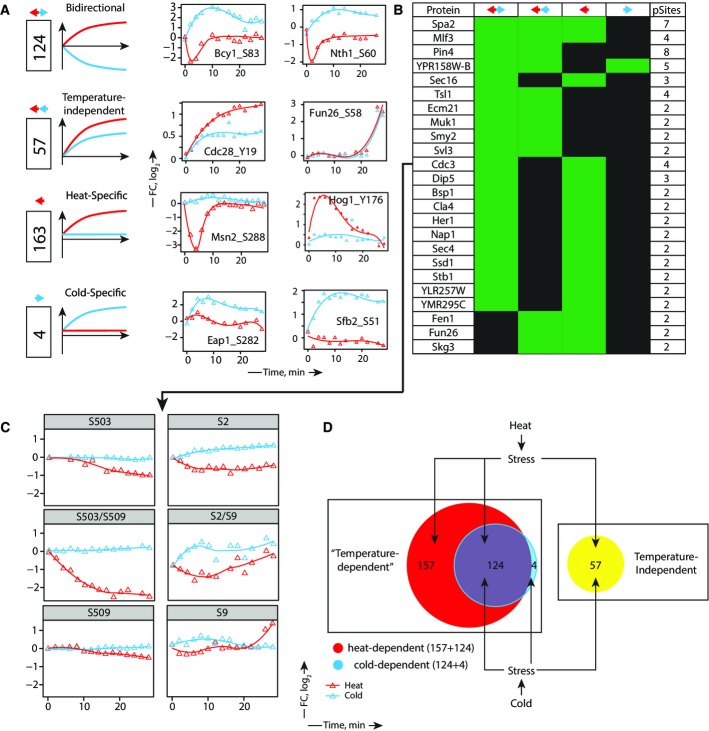
Cross-talk of signaling pathways upon heat and cold stresses

We observed that a larger proportion of dynamic phosphosites were observed under heat shock compared to cold stress, indicating that cellular responses to these stimuli are inherently different. To study the differential regulation of signaling pathways activated under these environmental changes, we grouped all dynamic phosphosites into four categories based on their response to cold and heat. Accordingly, the behaviors of dynamic phosphosites were classified as bidirectional, temperature independent or heat and cold specific to distinguish situations where profiles exhibited opposite, same response or no response to either cold or heat (Fig6A). We found that 36% of dynamic phosphosites are modulated in response to both heat and cold (Fig6A). Among these sites, we noted that 69% displayed opposite trends in phosphorylation upon these stimuli and could correspond to sites important for the regulation of protein activity. An example of this is represented by the neutral trehalase Nth1, an enzyme that hydrolyzes trehalose to glucose to provide energy and assist in recovery from stress. Earlier reports indicated that trehalose levels rise in response to heat shock and confer thermotolerance in the fission yeast *Schizosaccharomyces pombe* (Ribeiro *et al*, 1997). Importantly, trehalose levels must be tightly regulated, as cells lacking Nth1 exhibit impaired recovery from heat shock (Lee *et al*, 2013). Our data suggest that phosphorylation of Nth1 might regulate its enzymatic activity to control intracellular levels of trehalose.

Dynamic phosphosites are classified into four groups according to their response to heat (red) and cold (blue) along with representative examples.

A total of 24 out of 270 dynamic proteins contained phosphosites from different groups highlighting a complex regulation of kinase/phosphatase network.

Phosphorylation dynamics of septin Cdc3. Residues S503 and S509 are substrates of Cdc28, and their change in phosphorylation is known to regulate efficient ring disassembly (Tang & Reed, 2002). Dephosphorylation of both mono- and diphosphorylated forms of the corresponding peptide was observed upon heat shock, consistent with the inhibition of Cdc28 (see A). Dynamic changes on all phosphopeptide forms of S2 and S9 is also observed upon both heat shock and cold stress.

Venn diagrams representing overlap between dynamic phosphosites associated with heat and cold stresses. Responses to heat and cold are partially redundant; both trigger temperature-independent and temperature-dependent changes.

Our population of phosphosites contained a subset representing 16% of all dynamic sites that react in a similar manner to both increase and decrease in temperature (Fig6A). These phosphosites are probably involved in general stress response whereby signaling events trigger similar changes in cellular activities upon detection of abnormal temperature conditions. Among sites that showed a temperature-independent behavior, we noted the catalytic subunit of Cdc28, a master regulator of mitotic and meiotic cell cycles that has multiple roles in the regulation of cell cycle transcriptional programs, chromosome duplication and segregation, spindle dynamics, lipid biosynthesis, membrane trafficking, polarized cell growth and morphogenesis. Inhibition of Cdc28 activity upon temperature stress via the phosphorylation on Y19 residue might be required to cause cell arrest and prevent cell proliferation until favorable cell growth conditions are restored (Booher *et al*, 1993; Keaton *et al*, 2007).

The largest group (46%) of dynamic phosphosites is represented under the category heat-specific response and included proteins such as transcription factor Msn2 and Hog1 kinase (Fig6A). The kinase Hog1 was previously shown to be activated upon thermal stress (Soufi *et al*, 2009). Transient changes in Hog1 activity may reflect a variation in the perceived turgor pressure upon thermal stress. It is noteworthy that cold stress near freezing conditions is also known to activate Hog1 and trigger glycerol production essential for freeze protection (Panadero *et al*, 2006).

The last group typifying cold-specific response is represented by four phosphosites from eIF4E-associated protein Eap1, the component of the cleavage and polyadenylation factor I (CFI) Rna14, the transposon Ty1-PR2 Gag-Pol polyprotein YPR158W-B and the component of the COPII vesicle coat Sfb2 (Fig6A). While Eap1 is known to bind eIF4E for protein translation, it may also affect the activity of GCN4 translation via a cross-talk mechanism involving TOR signaling (Matsuo *et al*, 2005). The role of Eap1 phosphorylation is not known, though TOR activation results in the phosphorylation of the mammalian homolog eIF4E-binding protein to prevent its binding to eIF4E (Cosentino *et al*, 2000). The second representative of this group is Sfb2, a component of the COPII coat involved in the anterograde transport of vesicles from the endoplasmic reticulum to the Golgi apparatus. However, the functional significance of Sfb2 phosphorylation on its activity remains unknown.

Phosphoproteins can harbor several dynamic phosphosites with different behavior in response to heat and cold, and our dataset contained 24 out of 270 dynamic phosphoproteins with multiple phosphosites from different categories (Fig6B). One of such proteins is Cdc3, a component of the septin complex that displayed both bidirectional (S2/S9) and heat-specific (S503/S509) dynamics. We observed both mono- and diphosphorylated peptides for Cdc3 (Fig6C). The septins comprise a family of filament-forming proteins ubiquitous in eukaryotic species. It has been suggested that phosphorylation of Cdc3 by the Cdc28 kinase on S503/S509 at the end of G1 may facilitate initiation of a new cell cycle by promoting disassembly of the obsolete septin ring from the previous cell cycle (Tang & Reed, 2002). We observe dephosphorylation of both S503/S509 residues consistent with the inhibition of Cdc28 activity. While Cdc28 is inhibited under heat shock and cold stress, the dephosphorylation of Cdc3 appears to be heat specific suggesting a more complex regulatory mechanism. Phosphorylation of S2/S9 residues follows an opposite trend in response to heat and cold, possibly suggesting the interaction of different kinases. NetworKIN predictions indicated that S2 can be phosphorylated by Rad53 while S9 is a putative substrate for SCH9/PKC1 kinases. Another interesting example of multisite phosphorylation is the RNA binding protein Pin4. This protein is involved in G2/M phase progression and is known to be phosphorylated in response to DNA damage (Pike *et al*, 2004). We identified 8 phosphosites on Pin4 that are located within structural domains that likely affect its binding to RNA (Roy *et al*, 2010) under thermal stress (Supplementary Fig S10). Altogether, these results indicate that phosphosites exhibit strikingly different behaviors in response to cold and heat, reflecting the cellular plasticity and signaling rewiring necessary for cell adaptation to adverse environment. Heat shock triggers a large number of specific phosphorylation events not observed under cold stress, whereas a drop in temperature can trigger a signaling machinery common to heat shock to promote interconversion of metabolic intermediates necessary for cell survival (Fig6D).

### Significance of Cdc28 tyrosine 19 phosphorylation during the heat-shock response

**Figure 7:**
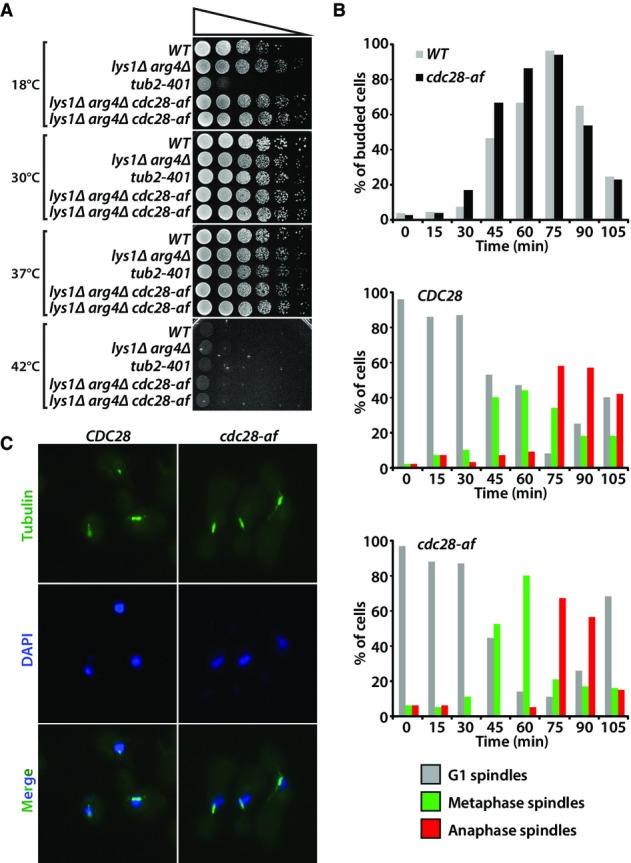
Cell cycle profile of *cdc28-af* mutants growing at elevated temperatures

Our phosphoproteomic data indicated that several substrates of the Cdc28 kinase become hypophosphorylated in response to heat shock. This decrease in phosphorylation correlates well with the increased phosphorylation at the inhibitory site Y19 of Cdc28. In vertebrate cells, loss of the equivalent phosphorylation on Cdk1 induces premature mitotic entry (Krek & Nigg, 1991), which suggests that Y19 phosphorylation in yeast could act by restraining cell cycle progression during the heat-shock response. To test this possibility, we constructed a *cdc28-af* mutant defective for Y19 and T18 phosphorylation and determined its cell cycle profile after heat shock. It was necessary to remove both T18 and Y19 phosphosites in Cdc28 since modification of the residue preceding the inhibitory tyrosine in CDKs can functionally compensate for the loss of tyrosine modification (Krek & Nigg, 1991; Sorger & Murray, 1992). *CDC28* and *cdc28-af* mutant cells were synchronously released from a G1 arrest into prewarmed medium, and samples were collected at regular intervals to determine the kinetics of budding and mitotic spindle formation. The release experiments were conducted at 30°C and 39°C because yeast cells did not cycle effectively at 42°C (Fig7A). Both *CDC28* and *cdc28-af* mutant cells formed buds and accumulated mitotic spindles with similar kinetics when released from a G1 arrest at 30°C (Supplementary Fig S11). In contrast, the appearance of cell cycle landmarks was advanced in the *cdc28-af* mutant released at 39°C relative to its wild-type counterpart (Fig7B and C). Specifically, *cdc28-af* cells showed an early accumulation of metaphase spindles at mitotic entry (60 min post-release; Fig7B and C) and faster kinetics of mitotic exit (as evidenced by an increased abundance of cells with G1/interphase-type microtubule arrays at the end of the experiment; Fig7B) when compared to *CDC28* cells growing at 39°C. These results are consistent with the known roles of Cdc28 in promoting bipolar spindle formation (Amon *et al*, 1993) and elongation (Rahal & Amon, 2008) during mitosis. Altogether, our results indicate that the increased phosphorylation of Cdc28 Y19 and associated reduction in kinase activity observed during the heat-shock response restrain cell cycle progression, most likely to allow time for cells to adapt to the cellular stress associated with increased temperatures and to promote effective cell division.

Growth properties of *CDC28* and *cdc28-af* mutant cells exposed to various temperatures. Fivefold dilutions of yeast cells were spotted on YPD plates and grown at 18, 30, 37 and 42°C for 2–4 days. Strains included on plates are as follows: D1 (WT), YALB6 (*lys1Δ arg4Δ*), D253 (*tub2-401,* a cold-sensitive strain), D4591 and D4592 (two independent clones with the *lys1Δ arg4Δ cdc28-af* genotype).

Budding index and spindle morphology of *CDC28* and *cdc28-af* cells after release from a G1 arrest into fresh medium at 39°C. Samples of cells were taken at intervals to determine the kinetics of bud appearance (top graph), and microtubule morphology (middle and bottom graphs) in cultures of *CDC28* and *cdc28-af* cells released synchronously into the cell cycle. At least 100 cells were counted at each time point.

Micrographs of *CDC28* and *cdc28-af* cells showing spindle morphology (tubulin) and nuclear mass position (DAPI) 60 min after G1 release at 39°C.

## Discussion

All organisms have evolved adaptation mechanisms to survive adverse environmental conditions such as suboptimal growth temperatures. In *S. cerevisiae*, the response and adaptation to heat shock and cold stress are initiated by complex and rapid signaling pathways that affect a wide range of cellular processes including transcriptional programs, cell division and morphogenesis, protein folding and metabolic changes in nutrient flux. Previous transcriptional profiling studies on the heat-shock response in yeast identified several differentially regulated genes involved in carbohydrate metabolism, protein folding and degradation, cytoskeletal reorganization, protein synthesis, ribosome synthesis and processing (Gasch *et al*, 2000; Gasch & Werner-Washburne, 2002). While our quantitative proteomics data indicated that only ~2% of proteins showed significant changes in protein abundance in the first 30 min upon heat shock, we noted that several proteins including HSP12, HSP26, HSP30, HSP42, HSP78, HSP82 and HSP104 were upregulated by at least two-fold. We also observed that a subset of ~0.4% of all quantified proteins including the mitochondrial electron transporter Cyc1 and Cdc25 that regulates Ras GTPase activity and cAMP cellular levels were downregulated following heat shock (Supplementary Table S3). We found that approximately half of the differentially abundant proteins from our proteomics data were also regulated in the previous transcriptomics studies (Gasch *et al*, 2000) and supported the activity of important regulatory mechanisms involved in response to environmental stress. However, the comparison of genes regulated upon heat shock with dynamic phosphoproteins identified in our study revealed an overlap of only 6% (119/1,959), suggesting that phosphorylation dynamics are not necessarily correlated with gene transcription and protein expression (Supplementary Table S8). These results also underline the significance of protein phosphorylation as a primary response to heat shock.

In the present study, we developed a quantitative phosphoproteomics workflow that enabled the first large-scale profiling of early signaling events in yeast upon heat shock and cold stress. The ability to monitor changes in protein phosphorylation with high temporal resolution facilitated the identification of regulated phosphosites based on the shape of kinetic profiles rather than conventional approaches that typically rely on FC measurements. This provided an inherent advantage in the identification of sites that displayed small but tractable changes in phosphorylation. A temporal resolution of 2 min was selected to minimize the occurrence of minima/maxima between measurements. In a recent study on osmotic shock response in yeast, we monitored changes in protein phosphorylation over a 1-min period with 5-s resolution and identified only monotonous profiles (Kanshin *et al*, 2015). Changes in phosphorylation including those associated with adaptation response likely take place over several minutes, and 2-min resolution was deemed optimal to detect these changes. The clustering of kinetic profiles enabled the grouping of new potential interactions between kinases and putative substrates, to further understand the integration of signaling pathways and the response to environmental stress.

Interestingly, we noted that an intricate network of kinases and phosphatases are rapidly activated upon changes in temperature. For example, deactivation of PKA subunit Tpk3 is taking place within 5 min upon heat shock and led to a transient dephosphorylation of several downstream substrates including Msn2/4 and Rgt1. The dephosphorylation of these transcription factors facilitates their nuclear import where they regulate the expression of genes associated with stress response element and different glucose transporters. Several other kinases and phosphatases displayed different temporal profiles as shown for Cdc28 and Mih1 which exhibited progressive and sustained changes in phosphorylation upon cell stimulation. The increased phosphorylation of Cdc28 at the inhibitory site Y19 was observed immediately upon changes in temperature and was mirrored by a dephosphorylation of known protein targets including Cdc14, Ace2 and Ste20. Several of these substrates play key regulatory roles during the progression through the cell cycle. The functional significance of Cdc28 inhibition during heat shock was also determined using *Cdc28-af* mutants where elevated temperatures resulted in advanced cell cycle landmarks compared to its wild-type counterpart. These results suggest that the reduced Cdc28 activity observed during heat-shock response slows down cell cycle progression and represents an adaptive response of yeast cells to this environmental stress.

We also noted that other proteins involved in cell cycle regulation also displayed important changes in phosphorylation. For example, Cdc14 is an important phosphatase that antagonizes Cdc28 activity during mitotic exit, and phosphorylation of Cdc14 S429 was recently reported to inhibit its activity during S phase (Li *et al*, 2014). During most of the cell cycle, Cdc14 is sequestered in the nucleolus where it associates with the nucleolar silencing establishing factor and telophase regulator, Net1, also referred to as Cfi1 (Straight *et al*, 1999; Visintin *et al*, 1999). In line with this, we identified nine phosphosites on Net1, of which two sites (S447, S497) showed kinetic profiles similar to those of Cdc28 substrates, consistent with a previous report indicating that dephosphorylation of both Cdc14 and Net1 favors their association in the nucleolus (Azzam *et al*, 2004).

Cdc28 is a master regulator of mitotic and meiotic cell cycles and associates with G1 (Clns), S and G2/M (Clbs) phase cyclins to direct substrate specificity. Eukaryotic cells rely on positive feedback in genetic control networks and cell cycle checkpoints to ensure proper transition across cell cycle phases. The start checkpoint corresponds to a short time interval in late G1 phase during which the yeast commits to division. In this period, the G1 cyclin Cln3 associates with Cdc28 and activates the transcription of about 200 genes by phosphorylating promoter-bound protein complexes such as transcription factors Sbf and Mbf1, and the transcriptional inhibitor Whi5 (Jorgensen & Tyers, 2004; Skotheim *et al*, 2008). We also observed that several phosphosites on Whi5 (S154, S156, S161) displayed kinetic profiles consistent with a decrease in Cdc28 activity, though these sites were not predicted by KinomeXplorer. Residues S154 and S156 are located within a nuclear export sequence (NES), and their dephosphorylation promotes the rapid nuclear translocation of Whi5 to inhibit SBF (Costanzo *et al*, 2004). Furthermore, Srl3 (S212), a Whi5 paralog, also showed a dephosphorylation profile at a consensus CDK site that reflected the reduced Cdc28 activity. Altogether, kinetic profiles obtained on Cdc28 substrates suggest that heat shock affects cell cycle regulation by preventing the G1/S transition and commitment to division.

Cell response to heat shock and cold stress was also reflected by an uneven distribution of dynamic phosphosites largely favoring the former stimulus. The observation that ~20% of all dynamic phosphosites were observed upon cold stress is consistent with lower enzymatic activities and metabolic rates expected in yeast at temperatures below optimal growth conditions. In contrast to heat shock, we only observed a small number of kinases and phosphatases that exhibit a change in phosphorylation upon cold stress. Interestingly, we noted a 1.4-fold increase in phosphorylation of Cdc28 at its inhibitory site Y19 during cold stress, but failed to observe a corresponding decrease in phosphorylation of Cdc28 substrates. This might suggest that a minimum activity threshold is required to detect changes in phosphorylation or that other regulatory mechanisms are involved. It is noteworthy that yeast cells can initiate different responses under cold shock conditions (10–18°C) compared to near-freezing temperatures (< 10°C) (Kandror *et al*, 2004). This possibly suggests that yeast can actively grow at 18°C, whereas growth is severely impeded at temperatures below 10°C. In spite of the low number of phosphosites regulated by the cold response, our quantitative phosphoproteomics analyses revealed that many of these sites were associated with the accumulation of carbohydrate reserves trehalose and glycogen. Indeed, we observed several phosphosites on Nth1 (S20, S21, S60, S66, S83), Tsl1 (S147, S161) and Gph1 (T31) that displayed opposite trends in response to cold and heat, possibly suggesting that phosphorylation regulates the activity of the corresponding enzymes to control intracellular pools of trehalose and glycogen.

While the present study provided valuable insights into early signaling events regulating biological functions in yeast under heat and cold stresses, we anticipate that improvements in both MS sensitivity and metabolic labeling reagents [e.g. neutron-encoded amino acids (Hebert *et al*, 2013)] will further expand phosphoproteome coverage and multiplexing capability of these experiments. The identification of putative substrates through the correlation of their kinetic profiles with those of their corresponding kinases opens interesting avenues for the selection of phosphosites of functional interest. Appropriate filters are required to select dynamic phosphosites displaying low variance and significant temporal changes. In the present study, we used polynomial fitting and selected only dynamic profiles with defined distribution (*N* ≥ 10) and high correlation coefficients (*R*^2^ > 0.9). Although the stringency of these selection criteria facilitated the selection of stimulus-specific profiles, we noted that phosphosites of functional relevance with fewer data points or higher variability of abundance can be excluded due to low intensity and/or poor ionization efficiency. This was the case for Hsf1 (S608, S655), a heat-shock transcription factor that exhibits temperature-dependent phosphorylation to initiate transcriptional programs associated with heat-shock elements (HSE) (Sorger & Pelham, 1988). Also, the phosphorylation profiles of some protein substrates might differ from those of their kinases due to the combined activity of other kinases or phosphatases for the same sites. The occurrence of complex phosphorylation profiles is time dependent, and phosphoproteomics experiments taking place over extended time periods are more likely to exhibit convoluted kinetic patterns. For example, Sic1 (T5) is a substrate and inhibitor of CDC28 and showed a dephosphorylation profile consistent with the change in Cdc28 activity for the first 20 min upon heat shock, but its return to basal levels at 28 min suggests a more complex regulation.

This contribution presents the first phosphoproteome dynamic study to investigate the response of yeast cells to rapid changes in temperature. Taken together, our data provide one of the most comprehensive phosphoproteomics study to identify differentially regulated signaling pathways upon heat shock and cold stress, and represent a unique resource to investigate cell biology paradigms associated with the control and regulation of these environmental stresses. We also anticipate that the tools and approaches described in this study can be applied to other cell stimuli to gain further understanding of the evolution and organization of signaling machineries.

## Materials and Methods

### Cell culture

Yeast strain S288C (*lys1Δ::kanMX; arg4Δ::kanMX*—a generous gift of Ole Jensen, University of Southern Denmark) was grown in synthetic dextrose (0.17% yeast nitrogen base without amino acids, 0.5% ammonium sulfate, 2% glucose and appropriate amino acids) supplemented with either light (Lys0/Arg0), medium (Lys4/Arg6) or heavy (Lys8/Arg10) isotopic forms of lysine (30 mg/l) and arginine (20 mg/l) (Cambridge Isotope Laboratories). The temperature was monitored throughout all experiments. The temperature of all SILAC cultures was 30°C (± 1°C) prior to heat or cold shock. Temperature stress experiments were achieved by adding 280 ml of fresh media at 30°C (control, light), 0°C (cold stress, medium) and 60°C (heat shock, heavy) to 175 ml of cell cultures maintained at 30°C to bring the final temperature at 30, 18 and 42°C, respectively. Flasks with cultures were immediately placed into temperature-controlled water bathes. Aliquots of 42 ml from each culture were collected every 2 min and combined in 700 ml of cold 95% ethanol (−80°C).

Yeast cells with an analog-sensitive mutant (*cdc28-as1*) were grown in the same media until OD_600_ = 0.7. Inhibition was performed by adding the pharmacological inhibitor 1NM-PP1 to a final concentration of 10 μM, whereas control cells were treated with vehicle (DMSO). After 15 min, TCA was added to final concentration 10% and cells were collected by centrifugation (2,000 × *g*, 10 min) and washed with ice-cold PBS. Proteins were extracted and digested as described below.

### Yeast mutant construction

To construct the *cdc28-as1* strain used for SILAC analysis, mutations resulting in the F88G change were introduced at the *CDC28* locus in strain YAL6B. The strain was transformed with a F88G—encoding PCR product amplified from plasmid p575, which carries the *CDC28::Tadh1::HIS3MX6* selection cassette (Ratsima *et al*, 2011). The presence of the F88G mutation (and the absence of secondary mutations) was confirmed by sequencing the entire *CDC28* open reading frame on chromosome 2 in strain D4485. To construct the *cdc28-af* mutant strain, plasmid p1288 was digested with PvuII, SalI and XhoI to release its *cdc28-T18A-Y19F::Tadh1::HIS3MX6* insert and transformed in strain YAL6B. Proper integration of *cdc28-af* mutations at the genomic locus was confirmed by selection on SC-HIS medium, PCR amplicon size analysis and sequencing the entire *CDC28* open reading frame in strain D4591.

### Protein extraction and enzymatic digestion

Cells were pelleted by centrifugation (2,000 × *g*, 10 min) and ethanol was discarded. Cell pellets were washed twice with 35 ml of cold (0°C) PBS and lysed by bead beating for 10 min in lysis buffer (8 M urea, 50 mM Tris pH 8.0, supplemented with HALT phosphatase inhibitor cocktail, Pierce). Samples were centrifuged at 40,000 × *g* for 10 min, and the supernatants were transferred into clean tubes prior to the determination of protein concentrations by BCA assay (Thermo Fisher Scientific). Disulfide bridges were reduced by adding dithiothreitol to a final concentration of 5 mM, and samples were incubated at 56°C for 30 min. Reduced cysteines were alkylated by adding iodoacetamide to 15 mM and incubating for 30 min in the dark at room temperature. Alkylation was quenched with 5 mM dithiothreitol for 15 min. Samples were diluted 6-fold with 20 mM TRIS, pH 8 containing 1 mM CaCl_2_ prior to overnight digestion at 37°C with trypsin (Sigma-Aldrich) using an enzyme to substrate ratio of 1:50 (w/w). Tryptic digests were acidified with 1% formic acid (FA), centrifuged (20,000 × *g* 10 min) and desalted on Oasis HLB cartridges (Waters). Peptide eluates were snap-frozen in liquid nitrogen, lyophilized in a SpeedVac centrifuge and stored at −80°C.

### Phosphopeptide isolation and fractionation

Tryptic digests were subjected to the TiO_2_ enrichment as described previously (Kanshin *et al*, 2013). Sample loading, washing and elution steps were performed using custom spin columns (Rappsilber *et al*, 2003; Ishihama *et al*, 2006) made from 200-μl pipette tip containing a SDB-XC membrane (3M) frit and filled with TiO_2_ beads. We equilibrate TiO_2_ material in 250 mM lactic acid in 70% ACN 3% TFA, and the same buffer was used for sample loading. Phosphopeptides were displaced from TiO_2_ with 500 mM phosphate buffer at pH = 7. Peptides were desalted in 50 μl of 1% FA and subsequently eluted from spin columns using 50 μl of 50% acetonitrile (ACN) 0.5% FA. Eluates were dried in a SpeedVac and stored at −80°C. To increase phosphoproteome coverage prior to MS analysis, phosphopeptides were fractionated offline by SCX chromatography. Peptides were solubilized in 100 μl of loading buffer (0.2% FA 10% ACN) and loaded onto StageTips containing 15 mg of poly-sulfoethyl-A SCX phase (5 μm 300A, Canada Life Science). Columns were washed with 50 μl of loading buffer, and peptides were eluted in six separate 100 μl salt steps of 15, 25, 50, 80, 150 and 1000 mM NaCl. Fractions were collected, dried in a SpeedVac and resuspended in 15 μl of 4% FA prior to MS analysis.

### Offline PGC fractionation of nonphosphorylated peptides

Peptides not retained on TiO_2_ resin (nonphosphorylated peptides) were diluted with water to reduce ACN concentration to 2% and subsequently desalted on Oasis HLB cartridges. Eluates were dried on a SpeedVac. Approximately 100 μg of peptides was fractionated offline on a PGC column (*Hypercarb*^*™*^ *Porous Graphitic Carbon,* 50 × 21 mm) using a 80-min ACN gradient from 0 to 80% in 10 mM ammonium formate, pH = 10 with a constant flow rate of 200 μl/min. Fractions were collected every 2 min and lyophilized on a SpeedVac. Peptides were resuspended in 50 μl of 4% FA and 10 μl was used for LC-MS/MS analyses.

### Mass spectrometry

Enriched phosphopeptide extracts were analyzed by LC-MS/MS using a Proxeon nanoflow HPLC system coupled to a Q-Exactive mass spectrometer (Thermo Fisher Scientific). Each sample was loaded on a reverse-phase precolumn (5 mm length, 360 μm i.d.) and separated on a reverse-phase analytical column (18 cm length, 150 μm i.d.) (Jupiter C_18_, 3 μm, 300 Å, Phenomenex). Both columns were packed manually. LC separations were performed at a flow rate of 0.6 μl/min using a linear gradient of 5–40% aqueous ACN (0.2% FA) in 120 min. MS spectra were acquired with a resolution of 70,000 using a lock mass (m/z: 445.120025) followed by up to 10 MS/MS data-dependent scans on the most intense ions using high energy dissociation (HCD). AGC target values for MS and MS/MS scans were set to 1e6 (max fill time 500 ms) and 1e6 (max fill time 120 ms), respectively. The precursor isolation window was set to m/z 2 with a HCD-normalized collision energy of 25. The dynamic exclusion window was set to 30s.

### Data processing and analysis

MS data were analyzed using MaxQuant (Cox & Mann, 2008; Cox *et al*, 2011b) software version 1.3.0.3 and searched against the SwissProt subset of the *S. cerevisiae* uniprot database (http://www.uniprot.org/) containing 6,630 entries. A list of 248 common laboratory contaminants included in MaxQuant was also added to the database as well as reversed versions of all sequences. The enzyme specificity was set to trypsin with a maximum number of missed cleavages set to 2. The precursor mass tolerance was set to 20 ppm for the first search (used for nonlinear mass re-calibration (Cox *et al*, 2011a) and then to 6 ppm for the main search. Phosphorylation of serine, threonine and tyrosine residues was searched as variable modification; carbamidomethylation of cysteines was searched as a fixed modification. The false discovery rate (FDR) for peptide, protein and site identification was set to 1%, the minimum peptide length was set to 6, and the “peptide requantification” function was enabled. The option match between runs (1-min time tolerance) was enabled to correlate identification and quantitation results across different runs. MaxQuant parameters.txt and experimentalDesign.txt files used in this study are listed in Supplementary Table S9. The mass spectrometry data from this publication have been submitted to the PeptideAtlas database (http://www.peptideatlas.org/) and assigned the identifier PASS00536 (password: EU474wm).

In addition to an FDR of 1% set for peptide, protein and phosphosite identification levels, we used additional criteria to increase data quality. First, we considered only peptides for which abundance ratios (FC = Stimulus/Control) were measured in at least 10 out of 15 time points. Then, we set a cutoff for average phosphosite localization confidence across experiments (time points) to 0.75. Final selection of dynamic profiles was achieved by fitting all temporal curves to a polynomial model and filtering out those fits with a regression coefficient *R*^2^ < 0.9 (Supplementary Fig S3 and Supplementary Table S2). Based on these criteria, we obtained 5,554 high-confidence profiles, of which 2,777 represented dynamic phosphosite profiles (Supplementary Table S1).

### Clustering of kinetic profiles, NetworKIN, GO and PPI network analysis

We used fuzzy C-means algorithm to cluster all dynamic profiles (Nock & Nielsen, 2006). Analysis and visualization were performed in the R environment (http://www.r-project.org/) with the Mfuzz package (Kumar & M, 2007). Optimal setting of the “fuzzifier” parameter was 1.235 as estimated with the “mestimate” function. In order to find the optimal number of clusters, we performed repeated soft clustering for cluster numbers ranging from 6 to 30 to calculate the minimum centroid distance (minimum distance between two cluster centers produced by c-means clustering) (Schwammle & Jensen, 2010). Prediction of kinase–substrate relationships was achieved using KinomeXplorer with default thresholding criteria (Horn *et al*, 2014) (http://networkin.info/). Gene ontology enrichment analyses were performed against whole *S. cerevisiae* proteome as background using DAVID bioinformatics resources for proteins containing dynamic phosphosites (Huang da *et al*, 2009a,b). A protein–protein interaction network was built in STRING for all proteins containing dynamic phosphosites (Franceschini *et al*, 2013). All interaction predictions were based on experimental evidences with the minimal confidence score of 0.9 (considered as a “highest confidence” filter in STRING). Results were visualized in Cytoscape network visualization and analysis (Shannon *et al*, 2003; Cline *et al*, 2007; Smoot *et al*, 2011).

### Yeast cell cycle analyses

Synchronization of cells in G1 was carried out using α-factor treatment (50 ng/ml for 120 min). Cells were released from the arrest by filtration and resuspension in fresh medium at the indicated temperatures. Samples were taken at 15-min intervals and processed to monitor bud formation and mitotic spindle morphology by indirect immunofluorescence, as previously described (Robellet *et al*, 2015). Spindle images were acquired on a DeltaVision microscope using the softWoRx software (Applied Precision). Conditions for serial dilution assays are as described previously (Robellet *et al*, 2015).

## Supporting Information

Supplementary Table S9

Review Process File

## References

[b1] Aguilera J, Randez-Gil F, Prieto JA (2007). Cold response in *Saccharomyces cerevisiae*: new functions for old mechanisms. FEMS Microbiol Rev.

[b2] Amon A, Tyers M, Futcher B, Nasmyth K (1993). Mechanisms that help the yeast cell cycle clock tick: G2 cyclins transcriptionally activate G2 cyclins and repress G1 cyclins. Cell.

[b3] Azzam R, Chen SL, Shou W, Mah AS, Alexandru G, Nasmyth K, Annan RS, Carr SA, Deshaies RJ (2004). Phosphorylation by cyclin B-Cdk underlies release of mitotic exit activator Cdc14 from the nucleolus. Science.

[b4] Bilsland-Marchesan E, Arino J, Saito H, Sunnerhagen P, Posas F (2000). Rck2 kinase is a substrate for the osmotic stress-activated mitogen-activated protein kinase Hog1. Mol Cell Biol.

[b5] Bodenmiller B, Wanka S, Kraft C, Urban J, Campbell D, Pedrioli PG, Gerrits B, Picotti P, Lam H, Vitek O, Brusniak MY, Roschitzki B, Zhang C, Shokat KM, Schlapbach R, Colman-Lerner A, Nolan GP, Nesvizhskii AI, Peter M, Loewith R (2010). Phosphoproteomic analysis reveals interconnected system-wide responses to perturbations of kinases and phosphatases in yeast. Sci Signal.

[b6] Booher RN, Deshaies RJ, Kirschner MW (1993). Properties of *Saccharomyces cerevisiae* wee1 and its differential regulation of p34CDC28 in response to G1 and G2 cyclins. EMBO J.

[b7] Budhwar R, Lu A, Hirsch JP (2010). Nutrient control of yeast PKA activity involves opposing effects on phosphorylation of the Bcy1 regulatory subunit. Mol Biol Cell.

[b8] Cline MS, Smoot M, Cerami E, Kuchinsky A, Landys N, Workman C, Christmas R, Avila-Campilo I, Creech M, Gross B, Hanspers K, Isserlin R, Kelley R, Killcoyne S, Lotia S, Maere S, Morris J, Ono K, Pavlovic V, Pico AR (2007). Integration of biological networks and gene expression data using Cytoscape. Nat Protoc.

[b9] Cosentino GP, Schmelzle T, Haghighat A, Helliwell SB, Hall MN, Sonenberg N (2000). Eap1p, a novel eukaryotic translation initiation factor 4E-associated protein in *Saccharomyces cerevisiae*. Mol Cell Biol.

[b10] Costanzo M, Nishikawa JL, Tang X, Millman JS, Schub O, Breitkreuz K, Dewar D, Rupes I, Andrews B, Tyers M (2004). CDK activity antagonizes Whi5, an inhibitor of G1/S transcription in yeast. Cell.

[b11] Cox J, Mann M (2008). MaxQuant enables high peptide identification rates, individualized p.p.b.-range mass accuracies and proteome-wide protein quantification. Nat Biotechnol.

[b12] Cox J, Michalski A, Mann M (2011a). Software lock mass by two-dimensional minimization of peptide mass errors. J Am Soc Mass Spectrom.

[b13] Cox J, Neuhauser N, Michalski A, Scheltema RA, Olsen JV, Mann M (2011b). Andromeda: a peptide search engine integrated into the MaxQuant environment. J Proteome Res.

[b14] De Wever V, Reiter W, Ballarini A, Ammerer G, Brocard C (2005). A dual role for PP1 in shaping the Msn2-dependent transcriptional response to glucose starvation. EMBO J.

[b15] Farrell A, Morgan DO (2000). Cdc37 promotes the stability of protein kinases Cdc28 and Cak1. Mol Cell Biol.

[b16] Ferreira JC, Silva JT, Panek AD (1996). A regulatory role for TSL1 on trehalose synthase activity. Biochem Mol Biol Int.

[b17] Franceschini A, Szklarczyk D, Frankild S, Kuhn M, Simonovic M, Roth A, Lin J, Minguez P, Bork P, von Mering C, Jensen LJ (2013). STRING v9.1: protein-protein interaction networks, with increased coverage and integration. Nucleic Acids Res.

[b18] Gancedo JM (2001). Control of pseudohyphae formation in *Saccharomyces cerevisiae*. FEMS Microbiol Rev.

[b19] Gasch AP, Spellman PT, Kao CM, Carmel-Harel O, Eisen MB, Storz G, Botstein D, Brown PO (2000). Genomic expression programs in the response of yeast cells to environmental changes. Mol Biol Cell.

[b20] Gasch AP, Werner-Washburne M (2002). The genomics of yeast responses to environmental stress and starvation. Funct Integr Genomics.

[b21] de Godoy LM, Olsen JV, Cox J, Nielsen ML, Hubner NC, Frohlich F, Walther TC, Mann M (2008). Comprehensive mass-spectrometry-based proteome quantification of haploid versus diploid yeast. Nature.

[b22] Gorner W, Durchschlag E, Wolf J, Brown EL, Ammerer G, Ruis H, Schuller C (2002). Acute glucose starvation activates the nuclear localization signal of a stress-specific yeast transcription factor. EMBO J.

[b23] Gruhler A, Olsen JV, Mohammed S, Mortensen P, Faergeman NJ, Mann M, Jensen ON (2005). Quantitative phosphoproteomics applied to the yeast pheromone signaling pathway. Mol Cell Proteomics.

[b24] Hebert AS, Merrill AE, Bailey DJ, Still AJ, Westphall MS, Strieter ER, Pagliarini DJ, Coon JJ (2013). Neutron-encoded mass signatures for multiplexed proteome quantification. Nat Methods.

[b25] Holly SP, Blumer KJ (1999). PAK-family kinases regulate cell and actin polarization throughout the cell cycle of *Saccharomyces cerevisiae*. J Cell Biol.

[b26] Holt LJ, Tuch BB, Villen J, Johnson AD, Gygi SP, Morgan DO (2009). Global analysis of Cdk1 substrate phosphorylation sites provides insights into evolution. Science.

[b27] Horn H, Schoof EM, Kim J, Robin X, Miller ML, Diella F, Palma A, Cesareni G, Jensen LJ, Linding R (2014). KinomeXplorer: an integrated platform for kinome biology studies. Nat Methods.

[b28] Hsieh YY, Hung PH, Leu JY (2013). Hsp90 regulates nongenetic variation in response to environmental stress. Mol Cell.

[b29] Huang da W, Sherman BT, Lempicki RA (2009a). Bioinformatics enrichment tools: paths toward the comprehensive functional analysis of large gene lists. Nucleic Acids Res.

[b30] Huang da W, Sherman BT, Lempicki RA (2009b). Systematic and integrative analysis of large gene lists using DAVID bioinformatics resources. Nat Protoc.

[b31] Ishihama Y, Rappsilber J, Mann M (2006). Modular stop and go extraction tips with stacked disks for parallel and multidimensional Peptide fractionation in proteomics. J Proteome Res.

[b32] Jacquet M, Renault G, Lallet S, De Mey J, Goldbeter A (2003). Oscillatory nucleocytoplasmic shuttling of the general stress response transcriptional activators Msn2 and Msn4 in *Saccharomyces cerevisiae*. J Cell Biol.

[b33] Jorgensen P, Tyers M (2004). How cells coordinate growth and division. Curr Biol.

[b34] Kandror O, Bretschneider N, Kreydin E, Cavalieri D, Goldberg AL (2004). Yeast adapt to near-freezing temperatures by STRE/Msn2,4-dependent induction of trehalose synthesis and certain molecular chaperones. Mol Cell.

[b35] Kanshin E, Michnick SW, Thibault P (2013). Displacement of N/Q-rich peptides on TiO2 beads enhances the depth and coverage of yeast phosphoproteome analyses. J Proteome Res.

[b36] Kanshin E, Bergeron-Sandoval LP, Isik SS, Thibault P, Michnick SW (2015). A cell-signaling network temporally resolves specific versus promiscuous phosphorylation. Cell Rep.

[b37] Keaton MA, Bardes ES, Marquitz AR, Freel CD, Zyla TR, Rudolph J, Lew DJ (2007). Differential susceptibility of yeast S and M phase CDK complexes to inhibitory tyrosine phosphorylation. Curr Biol.

[b38] Kim JH, Johnston M (2006). Two glucose-sensing pathways converge on Rgt1 to regulate expression of glucose transporter genes in *Saccharomyces cerevisiae*. J Biol Chem.

[b39] Krek W, Nigg EA (1991). Mutations of p34cdc2 phosphorylation sites induce premature mitotic events in HeLa cells: evidence for a double block to p34cdc2 kinase activation in vertebrates. EMBO J.

[b40] Kuhl NM, Rensing L (2000). Heat shock effects on cell cycle progression. Cell Mol Life Sci.

[b41] Kumar L, M EF (2007). Mfuzz: a software package for soft clustering of microarray data. Bioinformation.

[b42] Lee J, Reiter W, Dohnal I, Gregori C, Beese-Sims S, Kuchler K, Ammerer G, Levin DE (2013). MAPK Hog1 closes the *Saccharomyces cerevisiae* glycerol channel Fps1 by phosphorylating and displacing its positive regulators. Genes Dev.

[b43] Levy ED, Michnick SW, Landry CR (2012). Protein abundance is key to distinguish promiscuous from functional phosphorylation based on evolutionary information. Philos Trans R Soc Lond B Biol Sci.

[b44] Li Y, Cross FR, Chait BT (2014). Method for identifying phosphorylated substrates of specific cyclin/cyclin-dependent kinase complexes. Proc Natl Acad Sci USA.

[b45] Los DA, Murata N (2004). Membrane fluidity and its roles in the perception of environmental signals. Biochim Biophys Acta.

[b46] Matsuo R, Kubota H, Obata T, Kito K, Ota K, Kitazono T, Ibayashi S, Sasaki T, Iida M, Ito T (2005). The yeast eIF4E-associated protein Eap1p attenuates GCN4 translation upon TOR-inactivation. FEBS Lett.

[b47] Morano KA, Grant CM, Moye-Rowley WS (2012). The response to heat shock and oxidative stress in *Saccharomyces cerevisiae*. Genetics.

[b48] Nock R, Nielsen F (2006). On weighting clustering. IEEE Trans Pattern Anal Mach Intell.

[b49] Ong SE, Mann M (2006). A practical recipe for stable isotope labeling by amino acids in cell culture (SILAC). Nat Protoc.

[b50] Panadero J, Pallotti C, Rodriguez-Vargas S, Randez-Gil F, Prieto JA (2006). A downshift in temperature activates the high osmolarity glycerol (HOG) pathway, which determines freeze tolerance in *Saccharomyces cerevisiae*. J Biol Chem.

[b51] Pike BL, Yongkiettrakul S, Tsai MD, Heierhorst J (2004). Mdt1, a novel Rad53 FHA1 domain-interacting protein, modulates DNA damage tolerance and G(2)/M cell cycle progression in *Saccharomyces cerevisiae*. Mol Cell Biol.

[b52] Rahal R, Amon A (2008). Mitotic CDKs control the metaphase-anaphase transition and trigger spindle elongation. Genes Dev.

[b53] Raitt DC, Johnson AL, Erkine AM, Makino K, Morgan B, Gross DS, Johnston LH (2000a). The Skn7 response regulator of *Saccharomyces cerevisiae* interacts with Hsf1 *in vivo* and is required for the induction of heat shock genes by oxidative stress. Mol Biol Cell.

[b54] Raitt DC, Posas F, Saito H (2000b). Yeast Cdc42 GTPase and Ste20 PAK-like kinase regulate Sho1-dependent activation of the Hog1 MAPK pathway. EMBO J.

[b55] Rappsilber J, Ishihama Y, Mann M (2003). Stop and go extraction tips for matrix-assisted laser desorption/ionization, nanoelectrospray, and LC/MS sample pretreatment in proteomics. Anal Chem.

[b56] Ratsima H, Ladouceur AM, Pascariu M, Sauve V, Salloum Z, Maddox PS, D'Amours D (2011). Independent modulation of the kinase and polo-box activities of Cdc5 protein unravels unique roles in the maintenance of genome stability. Proc Natl Acad Sci USA.

[b57] Ribeiro MJ, Reinders A, Boller T, Wiemken A, De Virgilio C (1997). Trehalose synthesis is important for the acquisition of thermotolerance in Schizosaccharomyces pombe. Mol Microbiol.

[b58] Robellet X, Thattikota Y, Wang F, Wee TL, Pascariu M, Shankar S, Bonneil E, Brown CM, D'Amours D (2015). A high-sensitivity phospho-switch triggered by Cdk1 governs chromosome morphogenesis during cell division. Genes Dev.

[b59] Roelants FM, Torrance PD, Thorner J (2004). Differential roles of PDK1- and PDK2-phosphorylation sites in the yeast AGC kinases Ypk1, Pkc1 and Sch9. Microbiology.

[b60] Rowley A, Johnston GC, Butler B, Werner-Washburne M, Singer RA (1993). Heat shock-mediated cell cycle blockage and G1 cyclin expression in the yeast *Saccharomyces cerevisiae*. Mol Cell Biol.

[b61] Roy A, Kucukural A, Zhang Y (2010). I-TASSER: a unified platform for automated protein structure and function prediction. Nat Protoc.

[b62] Sadowski I, Breitkreutz BJ, Stark C, Su TC, Dahabieh M, Raithatha S, Bernhard W, Oughtred R, Dolinski K, Barreto K, Tyers M (2013). The PhosphoGRID *Saccharomyces cerevisiae* protein phosphorylation site database: version 2.0 update. Database.

[b63] Saleem RA, Rogers RS, Ratushny AV, Dilworth DJ, Shannon PT, Shteynberg D, Wan Y, Moritz RL, Nesvizhskii AI, Rachubinski RA, Aitchison JD (2010). Integrated phosphoproteomics analysis of a signaling network governing nutrient response and peroxisome induction. Mol Cell Proteomics.

[b64] Schade B, Jansen G, Whiteway M, Entian KD, Thomas DY (2004). Cold adaptation in budding yeast. Mol Biol Cell.

[b65] Schwammle V, Jensen ON (2010). A simple and fast method to determine the parameters for fuzzy c-means cluster analysis. Bioinformatics.

[b66] Shannon P, Markiel A, Ozier O, Baliga NS, Wang JT, Ramage D, Amin N, Schwikowski B, Ideker T (2003). Cytoscape: a software environment for integrated models of biomolecular interaction networks. Genome Res.

[b67] Skotheim JM, Di Talia S, Siggia ED, Cross FR (2008). Positive feedback of G1 cyclins ensures coherent cell cycle entry. Nature.

[b68] Smolka MB, Albuquerque CP, Chen SH, Zhou H (2007). Proteome-wide identification of *in vivo* targets of DNA damage checkpoint kinases. Proc Natl Acad Sci USA.

[b69] Smoot ME, Ono K, Ruscheinski J, Wang PL, Ideker T (2011). Cytoscape 2.8: new features for data integration and network visualization. Bioinformatics.

[b70] Sorger PK, Pelham HR (1988). Yeast heat shock factor is an essential DNA-binding protein that exhibits temperature-dependent phosphorylation. Cell.

[b71] Sorger PK, Murray AW (1992). S-phase feedback-control in budding yeast independent of tyrosine phosphorylation of P34cdc28. Nature.

[b72] Soufi B, Kelstrup CD, Stoehr G, Frohlich F, Walther TC, Olsen JV (2009). Global analysis of the yeast osmotic stress response by quantitative proteomics. Mol BioSyst.

[b73] Stark C, Su TC, Breitkreutz A, Lourenco P, Dahabieh M, Breitkreutz BJ, Tyers M, Sadowski I (2010). PhosphoGRID: a database of experimentally verified *in vivo* protein phosphorylation sites from the budding yeast *Saccharomyces cerevisiae*. Database.

[b74] Straight AF, Shou W, Dowd GJ, Turck CW, Deshaies RJ, Johnson AD, Moazed D (1999). Net1, a Sir2-associated nucleolar protein required for rDNA silencing and nucleolar integrity. Cell.

[b75] Tang CS, Reed SI (2002). Phosphorylation of the septin cdc3 in g1 by the cdc28 kinase is essential for efficient septin ring disassembly. Cell Cycle.

[b76] Urban J, Soulard A, Huber A, Lippman S, Mukhopadhyay D, Deloche O, Wanke V, Anrather D, Ammerer G, Riezman H, Broach JR, De Virgilio C, Hall MN, Loewith R (2007). Sch9 is a major target of TORC1 in *Saccharomyces cerevisiae*. Mol Cell.

[b77] Verghese J, Abrams J, Wang Y, Morano KA (2012). Biology of the heat shock response and protein chaperones: budding yeast (*Saccharomyces cerevisiae*) as a model system. Microbiol Mol Biol Rev.

[b78] Versele M, Thorner J (2004). Septin collar formation in budding yeast requires GTP binding and direct phosphorylation by the PAK, Cla4. J Cell Biol.

[b79] Visintin R, Hwang ES, Amon A (1999). Cfi1 prevents premature exit from mitosis by anchoring Cdc14 phosphatase in the nucleolus. Nature.

[b80] Walkey CJ, Luo Z, Borchers CH, Measday V, van Vuuren HJ (2011). The *Saccharomyces cerevisiae* fermentation stress response protein Igd1p/Yfr017p regulates glycogen levels by inhibiting the glycogen debranching enzyme. FEMS Yeast Res.

