# Supplementary material for: Phosphoproteome dynamics of Saccharomyces cerevisiae under heat shock and cold stress
Source: Mol Syst Biol. 2015 Jun 3;11(6):813. doi: 10.15252/msb.20156170 (PMC4501848; doi:10.15252/msb.20156170)
Supplement: Supplementary file 1 [file msb0011-0813-sd1.pdf]

Phosphoproteome dynamics of *Saccharomyces cerevisiae* under heat shock and cold stress

Evgeny Kanshin[1,5], Peter Kubiniok[1,2,5], Yogitha Thattikota[1,3], Damien D'Amours[1,3] and Pierre Thibault[1,2,4]*

[1]Institute for Research in Immunology and Cancer, Université de Montréal, C.P. 6128, Succursale centre-ville, Montréal, Québec, H3C 3J7, Canada.

[2] Department of Chemistry, Université de Montréal, C.P. 6128, Succursale centre-ville, Montréal, Québec, H3C 3J7, Canada.

[3] Department of Pathology and Cell Biology, Université de Montréal, C.P. 6128, Succursale centre-ville, Montréal, Québec, H3C 3J7, Canada.

[4] Department of Biochemistry, Université de Montréal, C.P. 6128, Succursale centre-ville, Montréal, Québec, H3C 3J7, Canada.

[5] These authors contributed equally to this work

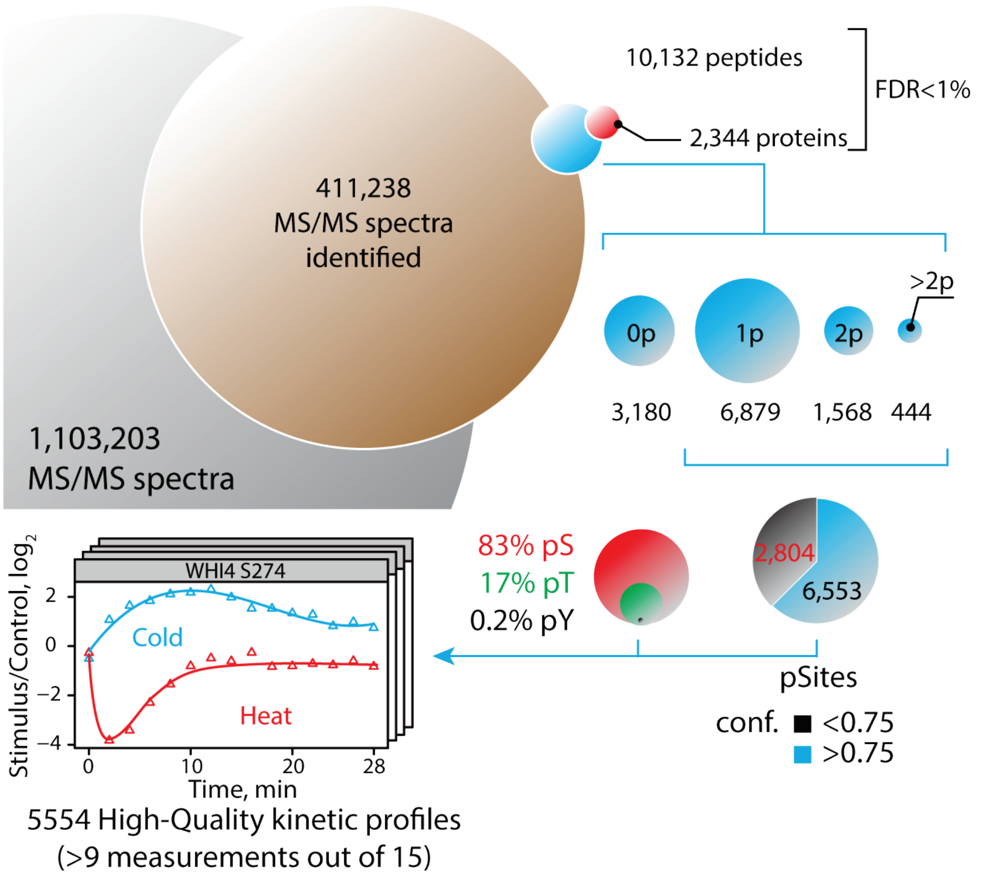

Figure S1. Summary of phosphopeptide identification. A total of 1,103,203 distinct MS/MS spectra were identified and assigned to 10,132 unique peptides from 2,344 proteins with a FDR < 1%. Phosphopeptides represented 73 % of all identified peptides, and 70% of all phosphosites (9,357) were assigned with high confidence (>0.75). A total of 5,554 dynamic profiles (≥ 10 out of 15 data points) were obtained from all high confidence assignments. Phosphorylation sites were distributed on Ser (83%) Thr (17%) and Tyr (0.2%) residues.

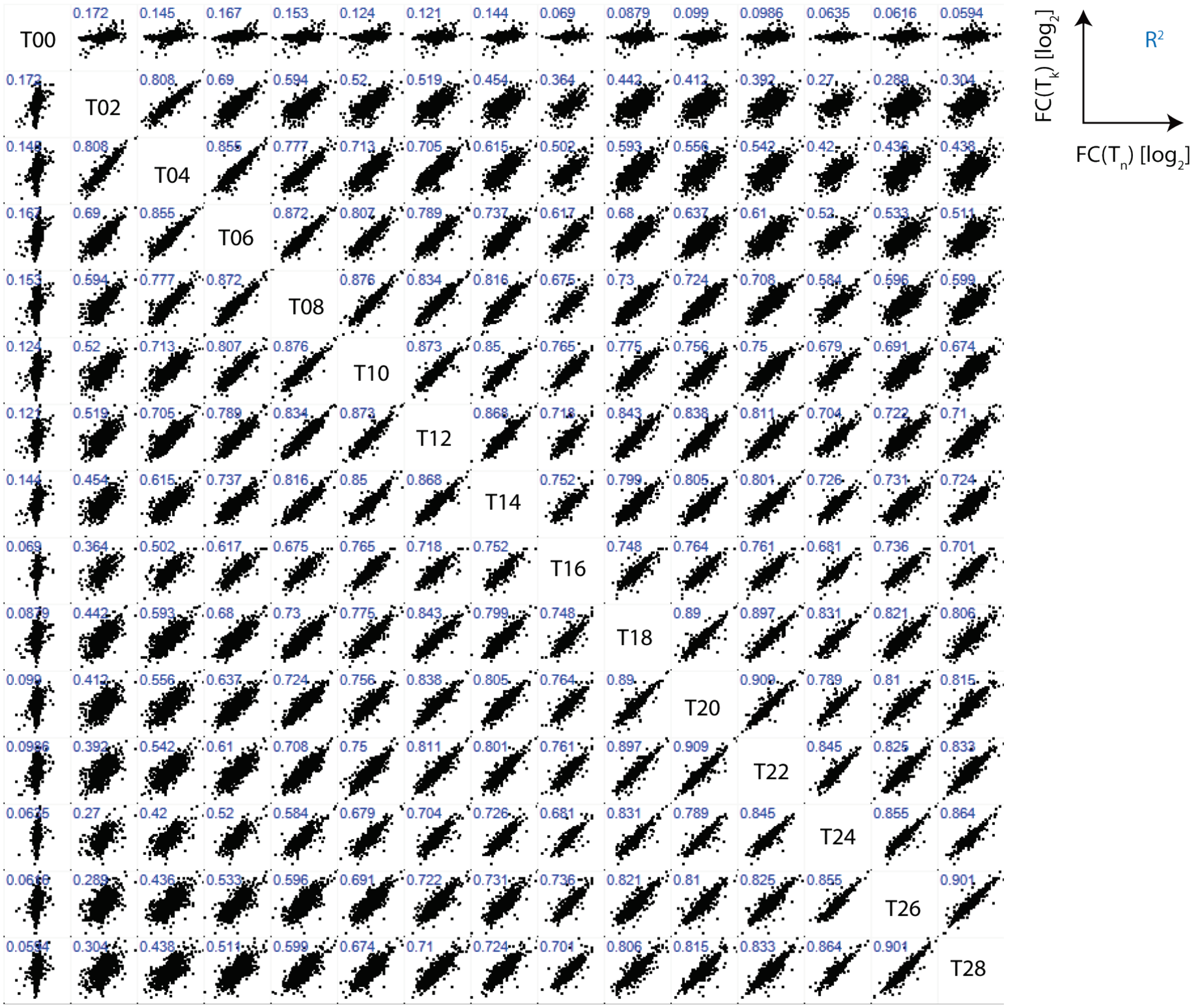

Figure S2: Pair wise comparison of phosphopeptide abundance. The comparison of SILAC ratios (H/L) is shown in a pair wise fashion for all time points corresponding to heat shock treatment. Phosphopeptide abundances of all SCX fractions are considered for each time point. R^2 values obtained are shown on the top left corner of each graph.

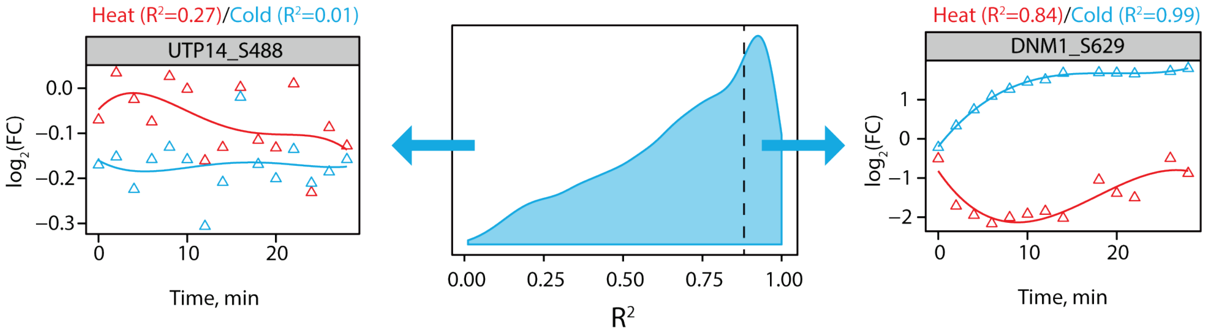

Figure S3. Finding regulated kinetic profiles in the dataset. In order to define subset of biologically regulated phosphosites, we performed fitting of all kinetic profiles using a polynomial model and selected only those with correlation coefficients (R^2) > 0.9 (middle panel). Representative examples of phosphosites with R^2 > 0.9 and R^2 < 0.9 are displayed.

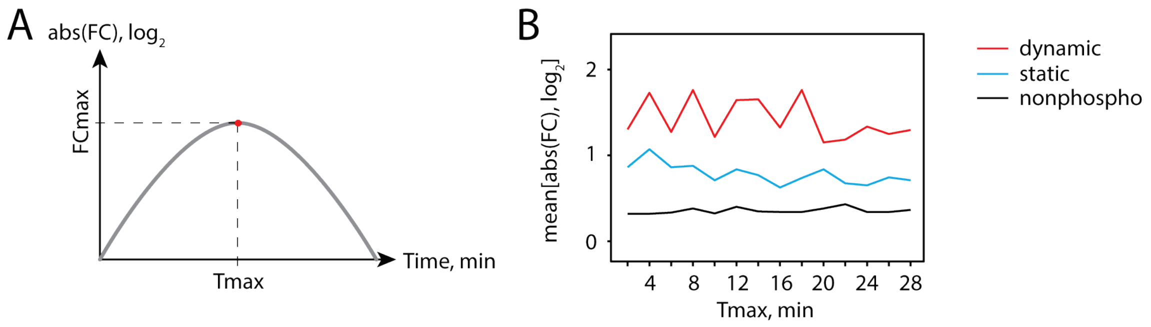

Figure S4. Amplitude of fold change (FC) for dynamic sites. A) From each profile the maximum amplitude (measured as absolute log_2-transformed FC was determined over the stimulation period. B) Mean values of the maximum amplitude at individual time points showing that dynamic sites displayed higher fold change compared to static sites or non phosphorylated peptides.

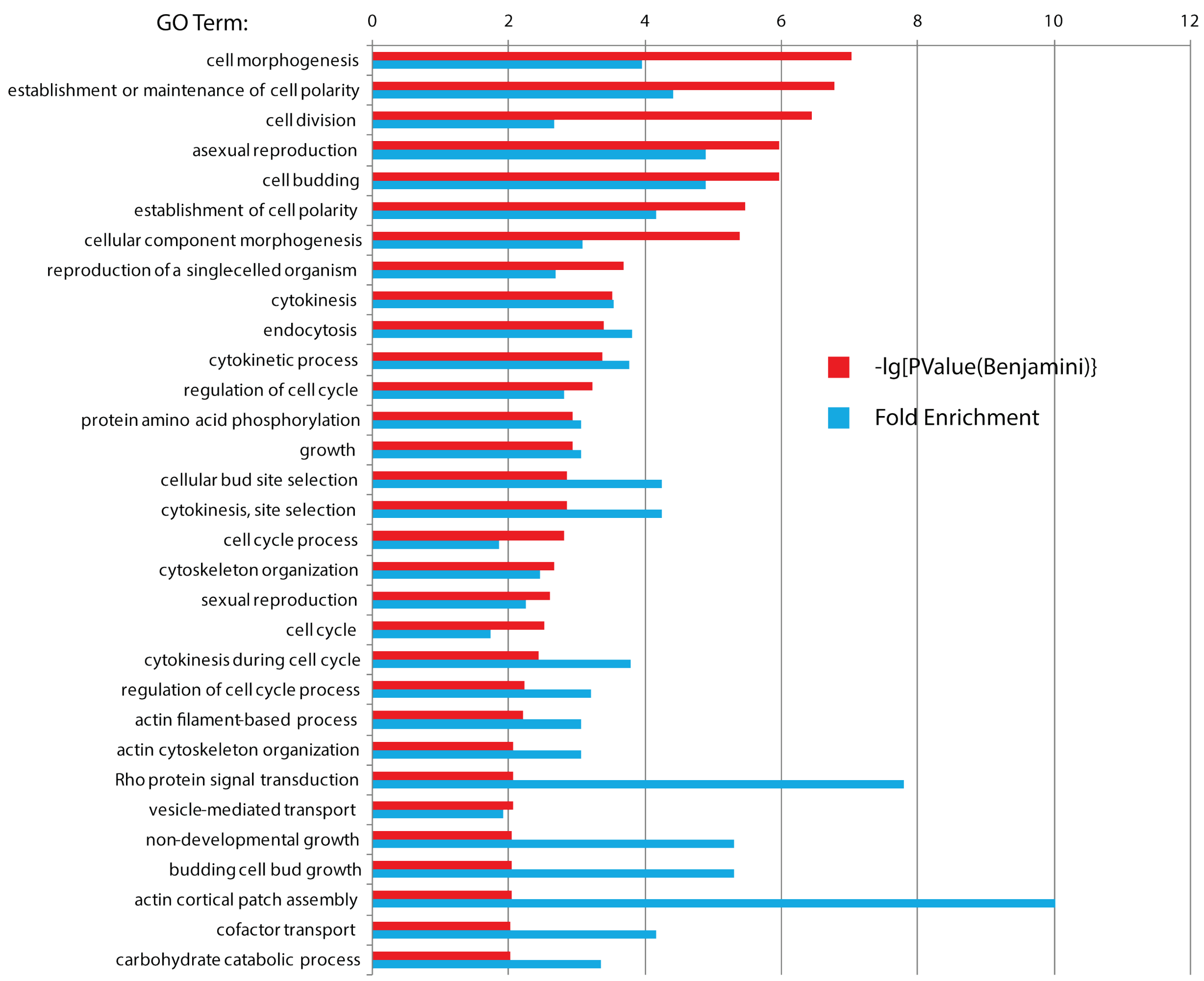

Figure S5. GO enrichment analysis for phosphoproteins showing dynamic phosphorylation upon heat shock and cold stress. Gene ontology enrichment analyses were performed using DAVID bioinformatics resources for proteins containing dynamic phosphorylation sites using the entire *S. cerevisiae* proteome as background. –Lg[PValue(Benjamini)] correspond to the negative log10 of pValue corrected for multiple hypothesis testing using Benjamini criteria.

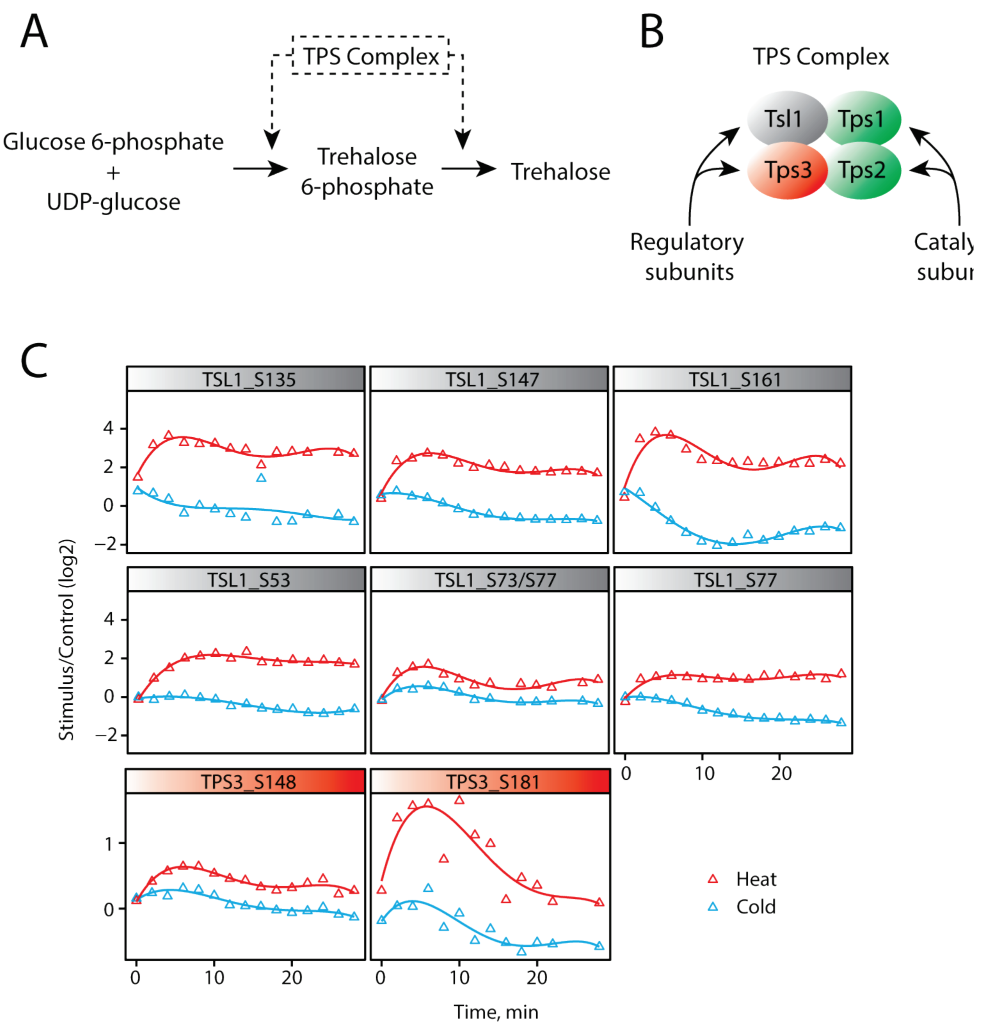

Figure S6. Dynamic changes in protein phosphorylation on the trehalose -6-phosphate synthase (TPS) complex regulatory subunit Tsl1. A) Trehalose biosynthesis involves a two step process whereby glucose 6-phosphate plus UDP-glucose are converted to trehalose 6-phosphate which is then converted to trehalose. Both steps are performed by the trehalose-6-phosphate synthase (TPS) complex. B) The yeast TPS complex comprises regulatory (Tsl1, Tps3) and catalytic (Tps1, Tps2) subunits. The Tps1 is associated with the conversion of glucose 6-phosphate into trehalose 6-phosphate while Tps2 subunit converts trehalose 6-phosphate into trehalose. Regulatory subunits Tps3 and Tsl1 have partially overlapping functions. C) Phosphorylation profiles of sites identified for the regulatory subunits Tsl1 and Tps3 following heat shock and cold stress.

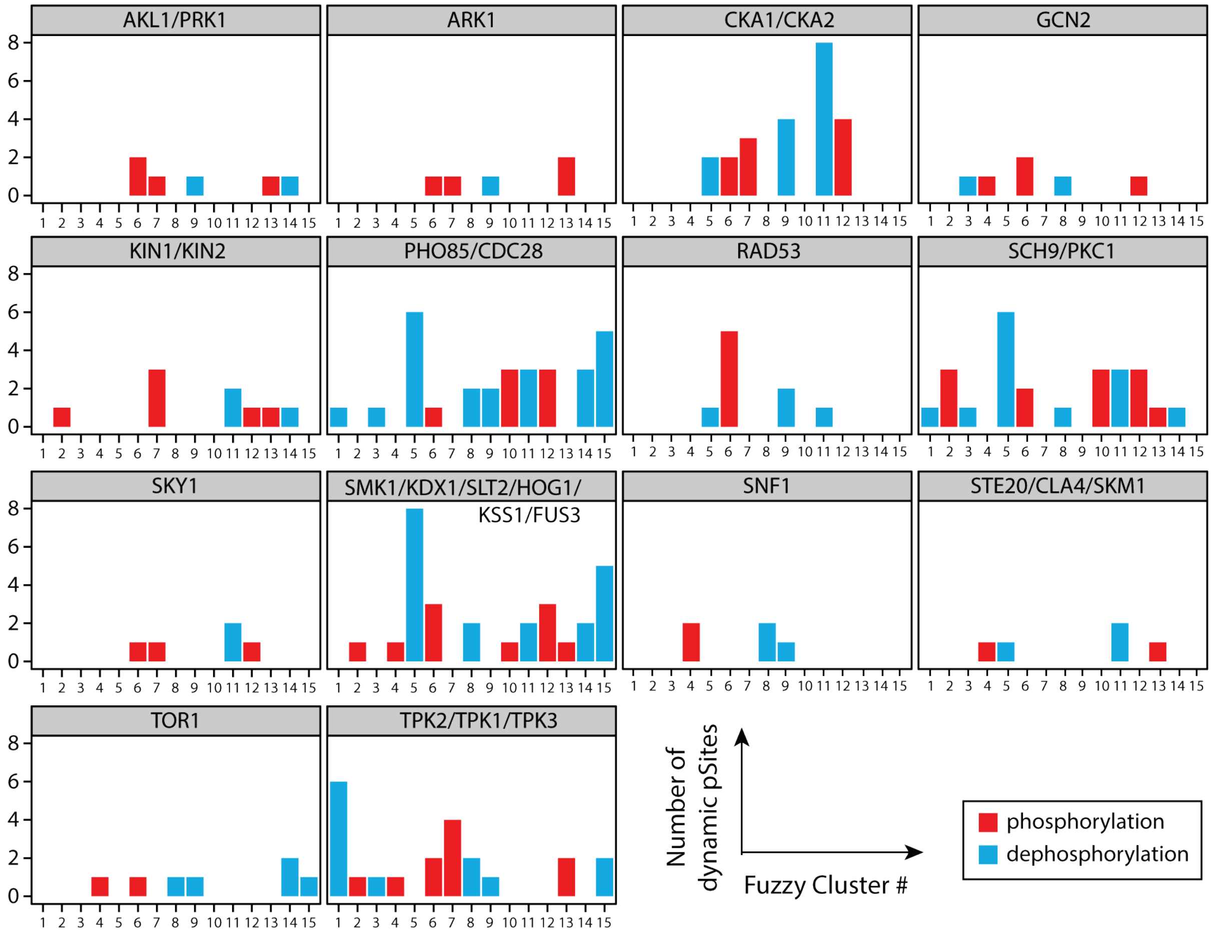

Figure S7. Distribution of dynamic behaviors among putative substrates of different kinase groups. We used KinomeXplorer to predict kinase groups associated with the phosphorylation of dynamic phosphosites detected in our study. Each panel represent the distribution of substrates for a particular kinase group for each fuzzy cluster (see Fig. 3), colors indicate whether the corresponding cluster represents an increase or a decrease in phosphorylation.

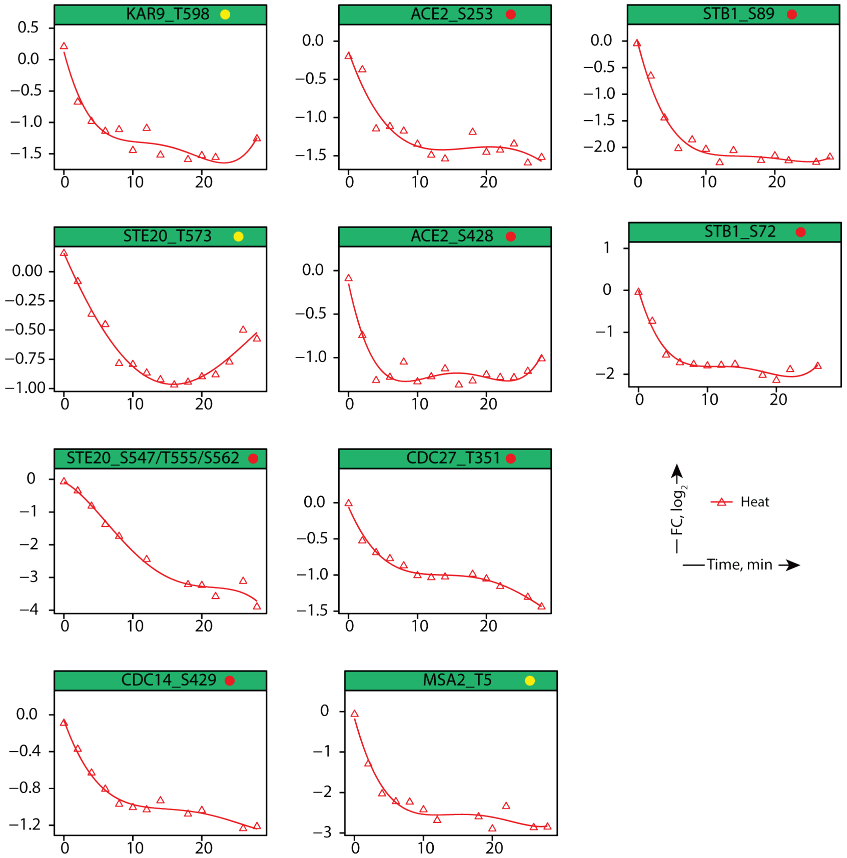

Figure S8. Dynamic behavior of Cdc28 substrates. An increase phosphorylation of Cdc28 at its inhibitory site Y19 was observed under in response to heat shock. STRING database was used to obtain high-confidence Cdc28 interactors among dynamic proteins detected in our study and PhosphoGRID database to infer known Cdc28-regulated phosphosites for these interactors. A total of 8 dynamic phosphosites (known substrates) were identified in our dataset, and all displayed similar dephosphorylation profiles (red dots). We detected similar dynamic profiles on phosphosites from other interactors, which were identified as putative substrates of Cdc28 (yellow dots).

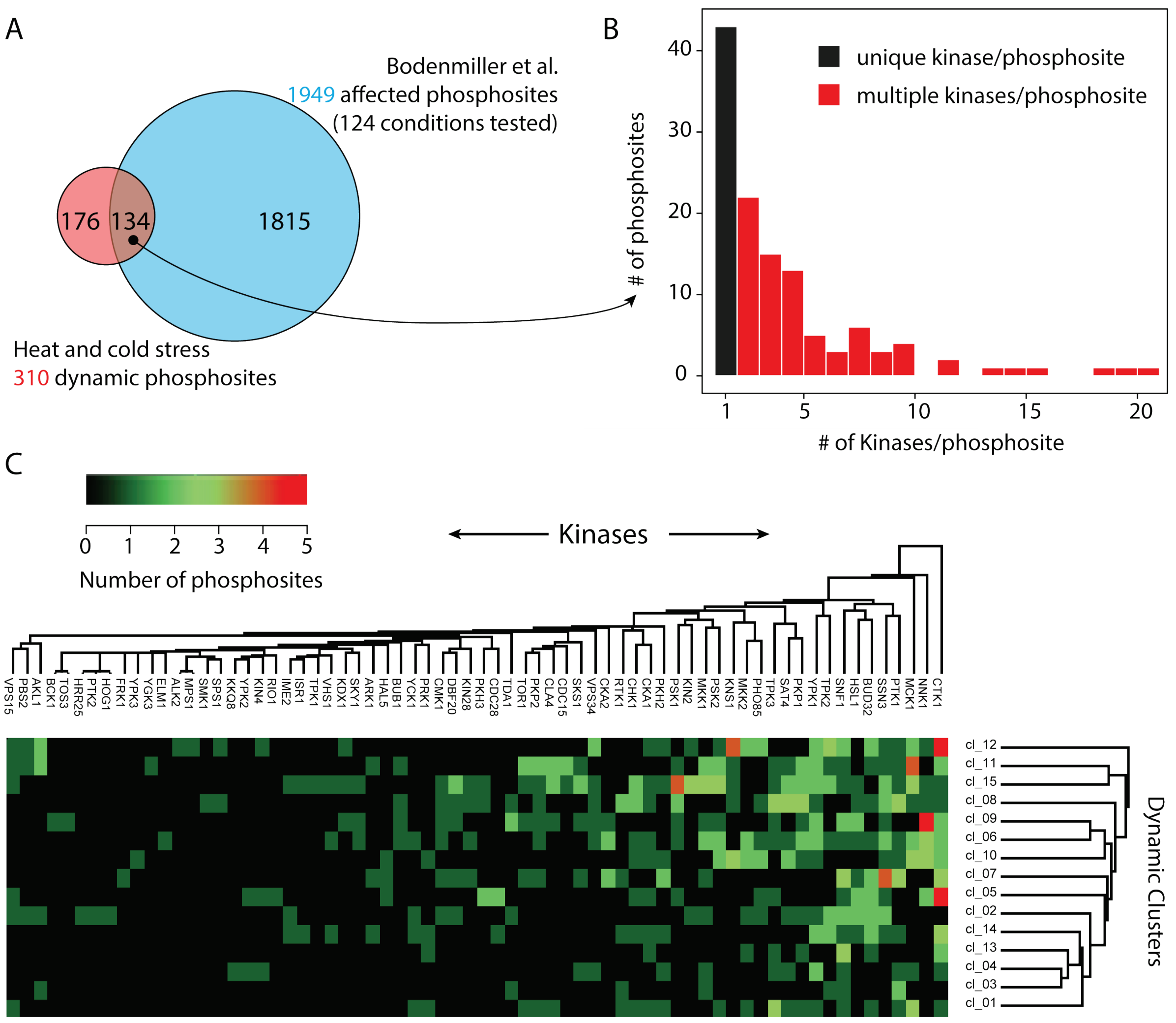

Figure S9. Overlap of dynamic phosphosites with system-wide perturbations of kinases and phosphatases in yeast. A) Comparison of dynamic phosphosites with those identified in large-scale phosphoproteomics study performed on 116 gene deletion mutants of the non essential kinases or phosphatases, and 8 analog-sensitive kinase strains of some essential kinases (Bodenmiller et al, 2010). Approximately 45% of dynamic phosphosites from monophosphorylated peptides (134 phosphosites) were also regulated in gene deletion or analog sensitive mutants. B) Distribution of dynamic phosphosites and their associated kinases. Majority of dynamic phosphosites are affected by more than one condition and associated with multiple kinases. C) Hierarchical clustering of kinases and their dynamic clusters. Heat map corresponds to grouping of individual kinases based on the dynamic behavior of the corresponding substrates.

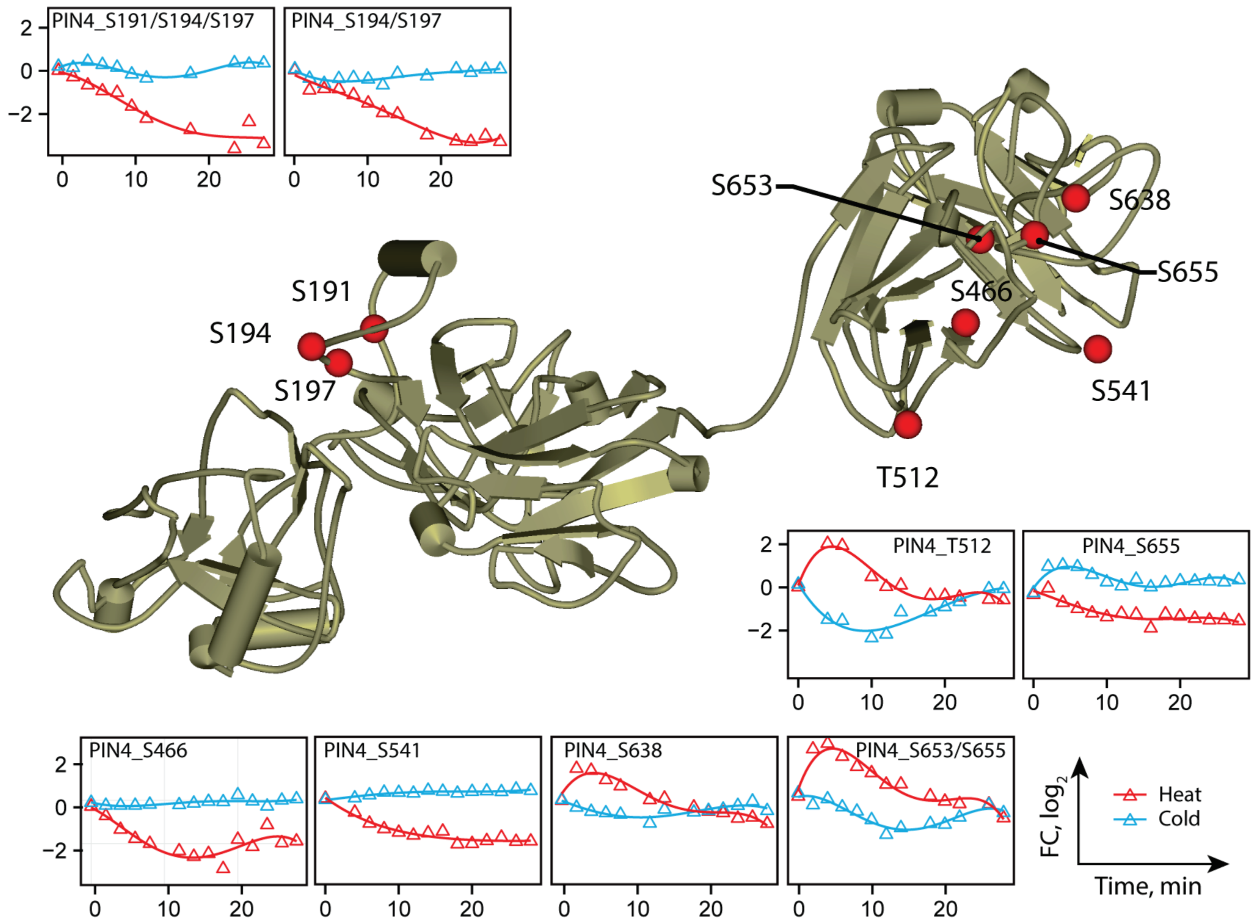

Figure S10. Distribution of dynamic sites within Pin4 structure. I-TASSER (Roy et al, 2010) was used to predict the structure of the RNA binding protein Pin4. Dynamic profiles of phosphosites are shown in response to heat and cold stresses. Changes in the phosphorylation of Pin4 could mediate its binding to RNA under changes of temperature.

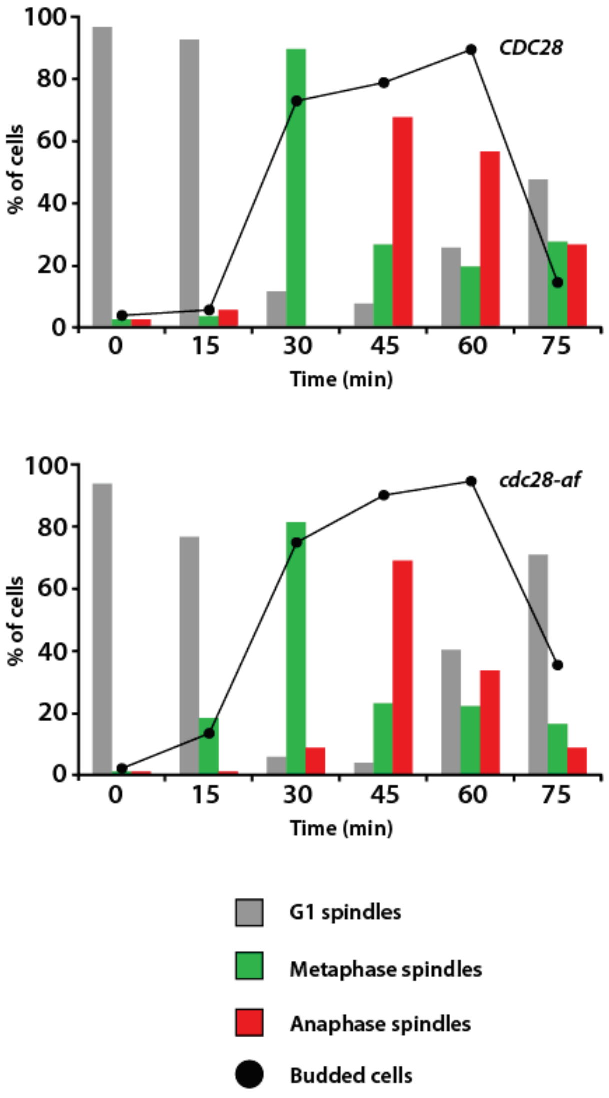

Figure S11. Cell cycle profile of *cdc28-af* mutants growing at physiological temperature. Graphs showing the budding index and spindle morphology of *CDC28* (top) and *cdc28-af* cells (bottom) after release from a G1-arrest into fresh medium at 30 °C. Sample of cells were taken every 15 min to determine the budding index (lines), and microtubule morphology (bars) in cultures of *CDC28* and *cdc28-af* cells progressing synchronously into the cell cycle. At least 100 cells were counted at each time point.
