# Supplementary material for: Phosphoproteome dynamics of Saccharomyces cerevisiae under heat shock and cold stress
Source: Mol Syst Biol. 2015 Jun 3;11(6):813. doi: 10.15252/msb.20156170 (PMC4501848; doi:10.15252/msb.20156170)
Supplement: Supplementary file 11 [file msb0011-0813-sd11.pdf]

molecular systems biology

Phosphoproteome dynamics of Saccharomyces cerevisiae under heat shock and cold stress

Evgeny Kanshin, Peter Kubiniok, Yogitha Thattikota, Damien D'Amours and Pierre Thibault

Corresponding author: Pierre Thibault, Universite de Montreal

Review timeline:

Submission date:	31 October 2014
Editorial Decision:	05 December 2014
Re-submission:	16 March 2015
Editorial Decision:	22 April 2015
Accepted:	18 May 2015

Editor: Thomas Lemberger

Transaction Report:

(Note: With the exception of the correction of typographical or spelling errors that could be a source of ambiguity, letters and reports are not edited. The original formatting of letters and referee reports may not be reflected in this compilation.)

1st Editorial Decision 05 December 2014

Thank you again for submitting your work to Molecular Systems Biology. We have now heard back from the three referees whom we asked to evaluate your manuscript. As you will see from the reports below, the referees raise substantial concerns on your work, which, I am afraid to say, preclude its publication in its present form.

While referee #1 is positive, the other two reviewers raise similar concerns as we initially expressed. While the quality of the dataset is universally acknowledged, both reviewer #2 and #3 note that further analysis and experimental follow up would be required to generate more concrete and novel biological insights.

As such, I am afraid that the level of support provided by the current reviewers remain too limited and I see no other choice than to return the manuscript with the message that we cannot offer to publish it.

Nevertheless, the editor and the reviewers expressed interest in your approach and clearly acknowledge the quality of your dataset, and we would not be opposed to consider a new and extended submission based on this work. Such an extended study should convincingly address the two following major directions:

- functional experimental investigations and a deeper computational analysis should be performed to provide significant biological insights, for example into how cells integrate signals.

- the potential confounding impact of cell growth should be disentangled from the effect of

temperature on signaling.

This would have a new number and receipt date. We recognise that this may involve further experimentation and analysis, and we can give no guarantee about its eventual acceptability. However, if you do decide to follow this course then it would be helpful to enclose with your re-submission an account of how the work has been altered in response to the points raised in the present review.

Thank you for the opportunity to examine this work.

Reviewer #1:

This publication provides a detailed view of changes in the phosphorylation pattern in the yeast S. cerevisiae exposed to sudden heat or cold exposure. The experimental approach relies heavily on current state of the art mass spectrometry with SILAC labeling for quantification, sample preparation and enrichment by multi-dimensional chromatography, leading edge data acquisition and interpretation followed by advanced bioinformatic tools. Perhaps the only novelty and main merit in the approach taken by the authors is high temporal resolution and number of samples taken during the course of the experiment. This strategy had the quite positive effect that the data are highly reliable and informative something that cannot always be said of previous publications related to studying changes in environmentally controlled protein phosphorylation patterns. Thus all together this work not only sets a high technical standard for similar studies but also provides a very useful resource for future projects related to how cells react and adapt to environmental stresses.
In my view, this study is also sufficiently extensive and presented well enough that one might safely refrain from any critical assessments.

Reviewer #2:

Summary: In this manuscript, the authors investigate the early signaling events that occur, in budding yeast, after either heat or cold shock at the level of protein phosphorylation. The molecular processes involved in the response to these stresses are still largely unknown. Complementing the transcriptional results already generated in the same system, the authors measured the phosphoproteome changes, induced by heat/cold shock, by shotgun mass spectrometry with SILAC labeling, thus providing a novel perspective of the processes involved, particularly the signaling cascades involved in the cell's response to temperature stress.

General comments: The paper contains a unique and high quality phospho-proteome dataset with a high temporal resolution, detailing the response of multiple phospho-sites to heat and, to a much lesser extent, cold shock in budding yeast. Such data is potentially rich in novel information: which proteins are involved in the signaling response, and the dynamics by which the response of each protein takes place. The dataset is the core of the paper and its main strength. The main focus of the authors is to use their dataset to hypothesize novel kinase-substrate interactions. The tools they use to find such interactions are not new and the predictions are not further verified. The data analysis part of the paper therefore consists, for the most part of a number of predictions arising from the data. Some of the observations are interesting and intriguing These include the observation that a much larger proportion of phospho-proteins respond to heat rather than cold shock, the involvement of several biological functions such as protein folding and degradation, regulation of cell cycle and cytokinesis, transcription regulation, morphogenesis in the process, the regongition that several kinases and phosphatases appear to respond to either of the two temperature stresses and predictions of predict novel kinase-substrate interactions. These do not, however, significantly add to the knowledge of how budding yeast respond to temperature stress, and how it deals with signaling integration in general. Addressing such questions experimentally or with more meaningful computer-aided methods would be logical next steps.

In its present form the paper does not provide a sufficient degree of new and validated biological insights to warrant publication in MSB.

In addition to these general issues there are also some concerns regarding the data analysis and the

way the work has been presented in the manuscript. Also, the language of the paper could be improved.

Major points
1. The dataset provided with this manuscript is high-throughput and time-resolved. While the first feature allowed them to identify which proteins and, specifically, which phospho-sites are involved in the response to heat/cold shock, the dynamic curves they compute for each site provide a further level of information. The authors employ clustering, first, to assess which phospho-sites are "dynamic" (responding to the stimulus) or "static" (not responding); second, to see if phosphosites associated to the same putative kinase have the same dynamic. Given the high temporal-resolution of their data, they should especially make further use of clustering techniques. For instance, they can see which responses take place before others by looking at their transitory phase: depending when a phosphosite reaches its highest/lowest point, it is possible to speculate on the timing of its activation/deactivation, on its location in the chain of signal transmission. The information in the dataset is therefore only exploited to a moderate degree. Further, the authors should explore the implications of including phosphopeptides observed in 10 out of 15 measurements. Are the conclusions reached robust to this arbitrary selection or would different thresholds (e.g. 15/15 or 8/15 to different conclusions?

2. Their high quality dynamic and quantitative data provided by the authors should be used to perform a network inference analysis via mathematical modeling tools (ODE equations, Bayesian networks...). Given how this methods can be quite time consuming, they could be restricted to a relatively small subset of interesting proteins. This would provide novel biological knowledge of a quality not achieved by the present very superficial computational analyses, and a starting point for future investigations on temperature stresses and on other cell stimulation studies.

3) The authors state that the same processes have been investigated by transcript analysis in the past. These data are not further discussed. It would be interesting to relate the present data to the transcript data or to at least relate the conclusions.

4) The whole discussion section is weak. For the most part is restates the findings of the results section without achieving a more in depth discussion by relating their data to other datasets and the present knowledge in the field.

5) The notion that concordance in the temporal profiles is biologically meaningful, in particular for the identification of kinase-substrate relationships is very shaky. The authors remark that themselves on page 10 in the discussion of the clusters. This questions some of the major conclusions and minimally, the basis for the assumption that temporal concordance has functional meaning should be discussed and rationalized. It is a well known fact that kinase substrates have different affinities for kinases and that this is reflected in different kinetic profiles.

Minor points
Abstract
1. The authors should highlight more biological findings, specifying for instance suggested novel kinase-substrate connections that they could find and that are interesting in the context of temperature shock response. The second sentence contains unnecessary details on transcriptome potential, which is a method they did not use and which is not further discussed.

Introduction
2. The authors should provide a clear scheme with all the knowledge that is already available from transcriptome profiling studies. In particular, this should include the proteins that will be mentioned in the rest of the manuscript (PKA, Cdc28, Sch9...). Which pathways? Receptors? Kinases? Signaling cascades? Transcription factors? This would greatly ease the understanding of the analyses they perform and the conclusions they draw, which involves many components that participate in several cellular functions.
3. First paragraph. They should provide more references. For instance: Aguilera Jaime 2007, Hoffmann Sara 2012, ...
4. Second paragraph. Sentences 2 to 5 are too verbose, and provide information that is only partially relevant to the method they've used. It would be better to shorten it, and give higher focus to the highlights of the paper. At the end of the paragraph they state "We reasoned that the acquisition of

high resolution kinetic profiles could provide valuable information not only on phosphoproteome dynamics but also on the correlation of kinase-substrate relationships." As discussed above, the basis for this statement should be minimally rationalized, and given the complex cluster membership also experimentally tested.

Results
Section "High temporal resolution of the S. cerevisiae phosphoproteome dynamics in response to heat and cold"
5. How many SCX fractions did they use?
6. Add SILAC protocol reference, either here on in Methods section
7. Did the author check the temperature before the first sampling?
8. Table E1 (file named "62904_0_table_797373_njbk60") should be more self-explanatory. No Sheets should be left without name. What is the content of Sheet1, Sheet2, Sheet3 and Sheet4? What is the meaning of the "Score" columns in the "Phosphopeptides" sheet?
9. How did the SILAC scatter plot look like? A few sample scatter plots should be added to supplementary material
10. The authors should explain how to interpret SILAC fold change ratios for readers that have no experience in the method.
11. Please explain why you chose to also plot interquartile ranges, what info they provide.
12. Why show the results for non-phosphorylated peptides? The samples were TiO2 enriched, so the data relative to these peptides is not at all meaningful or reliable.
13. (related to previous point) The authors state that "the abundance of non-phosphorylated peptides remained relatively constant over the entire time period of the experiment". This is not relevant and misleading, as one would expect the non-phosphorylated form of a peptide to have an opposite trend with respect to the phosphorylated one.
14. Third paragraph: it's not clear and needs rephrasing. How did you check for LOW VARIANCE? Explain why it can be important to also consider LOW AMPLITUDE profiles.
15. Why did you perform the fuzzy clustering here? What's its purpose?
16. Did you set some kind of threshold to distinguish between "static" and "dynamic" profiles? Or how did you do it?
17. Figure E4 (end of third paragraph) is called before Figure E3.
18. Please comment on the results you obtain, with a focus on general and temperature stress responses - the GO categories (can you provide some sample proteins from the main categories, and comment on them?), the kinases and phosphatases you find to be regulated (they are not too many).

Section "Temporal phosphorylation profiles identify potential kinase substrates in response to heat shock and cold stress"
19. This section is very long and it includes several different analyses. It would be much better to divide it into smaller sections, each one with a clear and progressive focus.
20. In line 6 it talks about FC, while at line 7 it says IQR. Are they both correct? From my understanding of the sentence, IQR should be FC.
21. Tsl1 and TSP should be mentioned in the introduction, as their involvement in heat response was previously known. Your result, which supports the reliability of your data, would not be easily registered and remembered.
22. Second paragraph. This paragraph needs to be restructured. First observe that in the case of Cdc28 Y19 (inhibition phospho-site) you noticed how its known targets have a specular behavior. Then suggest that, by looking at phospho-sites that cluster with together under the same putative kinase group, you may therefore be able to find novel targets of certain kinases. I would therefore start with the Cdc28 example, as it is more logical. The fact that phospho-sites of important kinases cluster in different groups doesn't necessarily point in the same direction.
23. Second paragraph. Why only the down-stream proteins?
24. Second paragraph. Please, better explain the meaning of a "putative kinase group".
25. Second paragraph. Please, provide a (supplementary at least) table with (indicatively) the following columns: phospho-site, protein, putative kinase group(s).
26. Second paragraph. The section that starts with "Next, we regrouped dynamic phosphosites associated to specific kinases..." needs a clearer explanation. If I got it right, it says that you checked if the phospho-sites that were found to be phosphorylated by the same putative kinases were grouped in the same cluster (had the same dynamic). Is this right?
26. Second paragraph. "... we also noted that several dynamic sites were predominantly observed in clusters of selected kinase groups" is not of clear interpretation. Do you mean to say that some

clusters/dynamics were more frequently represented, in several kinase groups?
27. Second paragraph. What happens if you group together the specular clusters (the ones that are already paired in Figure 5)? They could be treated as the same, in a way, as a dephosphorylation may induce an equivalent phosphorylation, for instance, if the dephosphorylation activated a kinase.
28. Third paragraph. This somehow belongs to the previous analysis of Cdc28, which should come before the rest.
29. Third paragraph. Can you comment on the results you obtain? What can you conclude from these results?
30. Fourth paragraph. Here the authors compared two very different datasets. What is the aim of this comparison?
31. Fifth paragraph. Msn2/4 were both already associated with heat shock, you should underline that.
32. Fifth paragraph. Please comment on the model shown in Figure 5. What is new about it?
33. Figure 5. Can you comment on the fact that most of the phospho-sites shown here are quickly and transiently DOWN-regulated by heat, while they are only mildly UP-regulated by cold? It's a very interesting and consistent behavior in response to both temperature stresses. And did you try to look at which phospho-sites cluster with Msn2/4?
34. Sixth paragraph. In this and other sections, the authors hypothesize a few novel interactions, which appear here and, sometimes, later in the text as they proceed with their investigation. This leads to confusion. Some of these could be used to generate a model involving several components, which could be used to suggest novel signaling processing mechanisms. You can try to provide a few more schemes like the one in Figure 5, at least those that you believe to be the most relevant.
35. Sixth paragraph. Can you comment on the fact that several glucose and glycogen metabolism enzymes are involved in the response? Was it expected or is it a surprise?

Section "Cross-talk of protein phosphorylation between heat shock and cold stress"
36. The conclusion to the first paragraph (the last sentence) is somewhat obvious. Can you speculate on its role during response to heat/cold shock?
37. Check the percentage you give in the second paragraph. Shouldn't it be 16% too be consistent with the other percentage you give in the rest of this section?
38. Did you also detect Hsp12? Hsp60? Hsp1? If so, what about them?
39. "Cdc28 is inhibited under heat shock and cold stress..." Can you give a reference to this fact?
40. The paper does not really address cross talk.

Discussion
40. You should highlight the importance of looking at the shape of a dynamic curve, and mention which information can be derived from it.
41. Can you comment on the choice of using a 2 minutes time resolution? How much would it be possible to reduce it in order to perform smaller experiments that still provide a similar amount of information - that is, without compromising the dynamic shapes you estimated from your measures?
42. Once again, do not only say that your study showed that by clustering the dynamic profiles you can hypothesize new interactions, but also and especially that with the data you have collected you could provide an advance in the knowledge on heat/cold shock response and on signaling integration mechanisms.
43. Can you comment on the fact that a much larger number of phospho-sites is responding to heat rather than cold shock? Why do you think that happened? Was the temperature not low enough?

Materials and Methods
44. Which protein:trypsin ratio did you use?
45. Phosphopeptide isolation protocol. Which buffers did you use for equilibration? And for elution?

Figures
46. Figure 2B. In the y-axis, is that IQR or log2FC? In the caption it talks about FC
47. Figure 2C. What's the meaning of the "node degree"?
48. Figure 5. Sch_S726 is missing a "9" (Sch9).
49. Figure 5. What's the meaning of the dotted connections? Which of these links were made or supported by your data?
50. Figure E3. What's -Ig[PValue(Benjamini)}?
51. Figure E6. It's not easy to understand which cluster the top-row bars correspond to. Also, what

do you mean by "distribution of substrates"?

Reviewer #3:

The present study revolves around the use of SILAC based phosphoproteomics to unravel the molecular responses to heat- and cold stress in the yeast Saccharomyces cerevisiae. The authors cultured an arginine and lysine auxothrophic yeast strain labeled with various stable isotopes of arginine and lysine in order to quantify relative differences in peptide levels after heat- or cold shock. Thus, at time point 0 the authors add media of different temperatures to induce temperature changes in the cultures and every second minute they harvest cells to examine the phosphoproteome by mass spectrometry after enrichment of phosphopeptides by TiO2 chromatography.

The study is technically very well executed and the strength of this study is clearly the large number of time points, which allow the authors to perform very thorough bioinformatics analyses. This analysis identify among others the cell cycle kinase Cdc28 and nutrient sensitive signaling kinases including PKA, TORC and Sch9 signaling as well as stress-regulated transcription factors Msn2/Msn4 as important regulatory circuits involved in the response to heat and cold stress.

Although this is a very interesting there are a number of issues that must be addressed.

Major points:
To induce the temperature shifts the authors add a large volume of cold or hot media, while the control media receives 30 degrees media. Although similar dilutions are induced of the cultures, it does not appear at what growth stage and to what density the cultures changes after dilution. Are the cells exponentially growing? Details in how cell culturing was carried out should be provided. Nonetheless, the volume added is larger than the volume of each culture resulting in at least halving the cell density. This means that the growth rate of the cells also change, and it must be expected that cells that are either cold or heat shocked do not grow, only the control cells will grow. This will inadvertently affect the conclusions that can be drawn from this study as the interpretation of the temperature change may be due to effects of growth. Therefore, the authors should show that the observed changes are caused by a change in temperature and not simply caused by a changed growth rate.

Moreover, as the main conclusions of the study seem to relate to Cdc28 functions and signaling related to nutrient sensitive pathways the effects of changes in temperature may just reflect a consequence of altered nutrient uptake, which affects cell cycle and hence Cdc28 activity, and hence not be an affect induced by temperature changes per se. Thus, how does the temperature changes affect nutrient uptake and metabolic fluxes e.g. incorporation of nutrients in to storage molecules and/or degradation of nutrients.

It does not appear if phosphopeptides have been normalized to the level of the total level of the same protein. Although it is the immediate response that has been examined, it cannot be excluded that protein levels are affected even after a few minutes. The authors should explain how each phosphorylated protein has been normalized.

Finally, the authors discuss the identified pathways and specific signaling components well in accordance with the literature although a few recent studies using SILAC based proteomics to examine starvation responses in yeast seem to have been omitted. However, none of their findings have been functionally validated, which certainly would increase the interest of the work. I do not expect the authors to examine the function of specific phosphorylation sites, but heat/cold shock responses should be examined in selected mutants carrying defects in relevant signaling pathways.

Minor points:
The authors should show in a suppl figure, which motif sequences that have been enriched in their analyses.

Re-submission 16 March 2015

Further to your messages of Dec. 3, we revised our manuscript (Phosphoproteome dynamics of Saccharomyces cerevisiae under heat shock and cold stress) and included additional experiments as you recommended in your decision letter. More specifically, you indicated that a new and extended submission should convincingly address the two following major directions:
(1) Functional experimental investigations and a deeper computational analysis should be performed to provide significant biological insights, for example into how cells integrate signals.
(2) The potential confounding impact of cell growth should be disentangled from the effect of temperature on signaling.
In agreement with our subsequent exchange of messages on Jan 17, we now include phosphoproteomics data on a Cdc28 (CDK1) analog sensitive mutant where the kinase was specifically inhibited by the pharmacological inhibitor 1NM-PP1. This allowed us to obtain an independent confirmation of substrates specific to Cdc28. These new results are in agreement with our previous assignments, and are now included in the new version of our manuscript. In addition, we conducted functional assays on a yeast strain where the WT Cdc28 was replaced by a Cdc28-af mutant defective for Y19 and T18 phosphorylation and determined its cell cycle profile after heat shock. These results indicated that the increased phosphorylation of Cdc28 Y19 and associated reduction in kinase activity observed during the heat shock response restrain cell cycle progression, to allow cells to adapt to the cellular stress associated with the increased temperature. We included these new data in our revised manuscript.
Reviewer 3 also raised the possibility that changes in protein abundance might be a confounding factor in the interpretation of regulation of phosphosites. Accordingly, we performed additional experiments on flow-through fractions containing non-phosphorylated peptides from TiO_2 enrichment experiments and quantified ~ 85 % from all our dynamic phosphoproteins (reported in Table E2). We observed that only in 2 of our dynamic phosphosites (Hsp42, Zeo1) showed changes in protein abundance higher than 2 fold, and these were excluded from the list of dynamic phosphoproteins.

As indicated in our previous cover letter, we believe that our data provide one of the most comprehensive phosphoproteomics study to identify differentially regulated signaling pathways upon heat shock and cold stress, and represent a unique resource to investigate cell biology paradigms associated to the control and regulation of these environmental stresses. We also anticipate that the tools and approaches described in this study can be applied to other cell stimuli to gain further understanding of the evolution and organization of signaling machineries.
We are planning to submit our phosphorylation data to the Phospho.ELM database and the Human Proteome Reference Database where these data can be consulted for the larger benefit of the cell signaling community. We transferred our files in Peptide Atlas.

Response to review of Manuscript (Dec. 3, 2014), "Phosphoproteome dynamics of Saccharomyces cerevisiae under heat shock and cold stress"
Italic highlights response on specific comments raised by the editor and the reviewers
***Bold** type indicate changes to the manuscript, also highlighted in yellow on the revised manuscript*

We are most grateful for the valuable comments of the editor and reviewers which improved the quality of our manuscript. We addressed every points raised as follow:
Editor's comment:
The editor and the reviewers expressed interest in your approach and clearly acknowledge the quality of your dataset, and we would not be opposed to consider a new and extended submission based on this work. Such an extended study should convincingly address the two following major directions:

(1) Functional experimental investigations and a deeper computational analysis should be performed to provide significant biological insights, for example into how cells integrate signals.

(2) The potential confounding impact of cell growth should be disentangled from the effect of temperature on signaling.

© European Molecular Biology Organization

This would have a new number and receipt date. We recognise that this may involve further experimentation and analysis, and we can give no guarantee about its eventual acceptability. However, if you do decide to follow this course then it would be helpful to enclose with your re-submission an account of how the work has been altered in response to the points raised in the present review.
Our revised manuscript which is now submitted as a new contribution contains new data addressing both points above. We addressed point 1 above by including phosphoproteomics data on Cdc28 analog sensitive mutant where the kinase can be inhibited specifically by the pharmacological inhibitor 1NM-PP1. This allowed us to obtain an independent confirmation of substrates specific to Cdc28. We made appropriate changes to our manuscript on p. 11 to account for these new results. We also complemented these studies with functional assays on a yeast strain where the WT CDC28 gene was replaced by a cdc28-af mutant defective for Y19 and T18 phosphorylation and determined its cell cycle profile after heat shock. These results indicated that the increased phosphorylation of Cdc28 Y19 and associated reduction in kinase activity observed during the heat shock response restrain cell cycle progression, most likely to allow time for cells to adapt to the cellular stress associated with increased temperatures and to promote effective cell division. These new data are now part of a separate section on p. 17 of the revised manuscript.
Regarding the possibility that changes in protein abundance might be a confounding factor in the interpretation of regulation of phosphosites (point 2) we performed additional experiments on flow-through fractions containing nonphosphorylated peptides from TiO_2 enrichment experiments. We desalted samples and performed offline HPLC fractionation on PGC (Hypercarb™ Porous Graphitic Carbon) column collecting 40 fractions that were subsequently analyzed by MS. These results are presented in Table E3).This allowed us to quantify ~ 85 % from all our dynamic phosphoproteins (reported in Table E2). We observed that only in 2 of dynamic phosphosites showed changes in protein abundance higher than 2 fold. Therefore HSP42 (log2(FC)=2.06) and ZEO1 (log2(FC)=1.33) were excluded from the list of dynamic phosphoproteins.

Reviewer #1:

This publication provides a detailed view of changes in the phosphorylation pattern in the yeast S. cerevisiae exposed to sudden heat or cold exposure. The experimental approach relies heavily on current state of the art mass spectrometry with SILAC labeling for quantification, sample preparation and enrichment by multi-dimensional chromatography, leading edge data acquisition and interpretation followed by advanced bioinformatic tools. Perhaps the only novelty and main merit in the approach taken by the authors is high temporal resolution and number of samples taken during the course of the experiment. This strategy had the quite positive effect that the data are highly reliable and informative something that cannot always be said of previous publications related to studying changes in environmentally controlled protein phosphorylation patterns. Thus all together this work not only sets a high technical standard for similar studies but also provides a very useful resource for
future projects related to how cells react and adapt to environmental stresses.
In my view, this study is also sufficiently extensive and presented well enough that one might safely refrain from any critical assessments.

Reviewer #2:

Summary: In this manuscript, the authors investigate the early signaling events that occur, in budding yeast, after either heat or cold shock at the level of protein phosphorylation. The molecular processes involved in the response to these stresses are still largely unknown. Complementing the transcriptional results already generated in the same system, the authors measured the phosphoproteome changes, induced by heat/cold shock, by shotgun mass spectrometry with SILAC labeling, thus providing a novel perspective of the processes involved, particularly the signaling cascades involved in the cell's response to temperature stress.

General comments: The paper contains a unique and high quality phospho-proteome dataset with a high temporal resolution, detailing the response of multiple phospho-sites to heat and, to a much lesser extent, cold shock in budding yeast. Such data is potentially rich in novel information: which proteins are involved in the signaling response, and the dynamics by which the response of each protein takes place. The dataset is the core of the paper and its main strength. The main focus of the

authors is to use their dataset to hypothesize novel kinase-substrate interactions. The tools they use to find such interactions are not new and the predictions are not further verified. The data analysis part of the paper therefore consists, for the most part of a number of predictions arising from the data. Some of the observations are interesting and intriguing These include the observation that a much larger proportion of phospho-proteins respond to heat rather than cold shock, the involvement of several biological functions such as protein folding and degradation, regulation of cell cycle and cytokinesis, transcription regulation, morphogenesis in the process, the recognition that several kinases and phosphatases appear to respond to either of the two temperature stresses and predictions of predict novel kinase-substrate interactions. These do not, however, significantly add to the knowledge of how budding yeast respond to temperature stress, and how it deals with signaling integration in general. Addressing such questions experimentally or with more meaningful computer-aided methods would be logical next steps.

In its present form the paper does not provide a sufficient degree of new and validated biological insights to warrant publication in MSB.
Additional data are now presented on Cdc28-as mutant to validate substrate assignment. We added an entirely new section on the significance of Cdc28 tyrosine 19 phosphorylation during the heat shock response as discussed above. More details are provided on these specific points below.

In addition to these general issues there are also some concerns regarding the data analysis and the way the work has been presented in the manuscript. Also, the language of the paper could be improved.
We revised the manuscript and provided whenever possible new data to clarify points raised by the reviewer.

Major points
1. The dataset provided with this manuscript is high-throughput and time-resolved. While the first feature allowed them to identify which proteins and, specifically, which phospho-sites are involved in the response to heat/cold shock, the dynamic curves they compute for each site provide a further level of information. The authors employ clustering, first, to assess which phospho-sites are "dynamic" (responding to the stimulus) or "static" (not responding); second, to see if phosphosites associated to the same putative kinase have the same dynamic. Given the high temporal-resolution of their data, they should especially make further use of clustering techniques. For instance, they can see which responses take place before others by looking at their transitory phase: depending when a phosphosite reaches its highest/lowest point, it is possible to speculate on the timing of its activation/deactivation, on its location in the chain of signal transmission. The information in the dataset is therefore only exploited to a moderate degree. Further, the authors should explore the implications of including phosphopeptides observed in 10 out of 15 measurements. Are the conclusions reached robust to this arbitrary selection or would different thresholds (e.g. 15/15 or 8/15 to different conclusions?
We agree with the reviewer regarding the importance of the temporal order of pSite activation. Our data suggest that all changes in protein phosphorylation start immediately after system perturbation. This makes it difficult to clearly order signaling events with time. We have also attempted to use transitory phase. Its definition is based on particular mathematical function used to fit dynamic profiles. The kinetic parameters obtained will be different depending on the function used for fitting profiles. Each dynamic profile arise from multiple factors (activities and concentrations of different kinases and phosphatases, etc…) and it is difficult to determine which fitting function would provide the best model. More importantly, several types of kinetic curves would probably require different mathematical models to perform fittings which further complicate these analyses. Another important consideration is relative nature of the SILAC quantification, we always obtain ratio of pSite abundance between control and perturbed conditions. This ratio does not reflect actual stoichiometry of phosphorylation therefore our data cannot be used to calculate actual enzymatic rates. Due to these factors, we were not able to come up with a robust algorithm to determine the temporal order of signaling events.
This reviewer also raised an important question regarding the number of measurements across time frame. Since our data analysis is based on the shape of the response, we need quantitative measurements across substantial number of time points to define a profile with high confidence. Obviously it would be ideal to consider only pSites quantified across all 15 time points but this would result in only ~7% of the data. In contrast, if we consider pSites with few measurements the

quality of the corresponding profiles would be compromised. The selection of "10 out of 15 measurements" was based on the visual inspection of the following distribution:

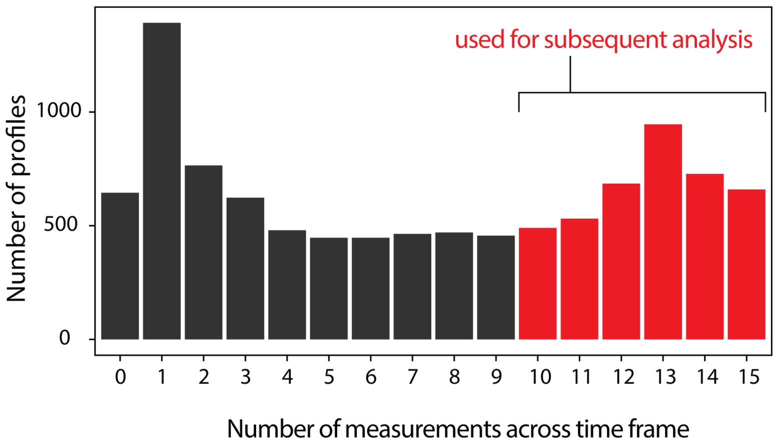

When selecting at least 10 measurements across the time frame of the experiment, we prioritize quality over the number of profiles. Since these profiles represent ~40% of the whole dataset we believe these correspond to a representative group of the whole dataset. We did not obtain new types of information (like a new signaling pathways or new enriched GO terms) by using less restrictive threshold criteria. Choosing 8/15 measurements would allow us to use ~50% (only 10% difference) of the dataset at the expense of quality. By using 15/15-profiles we would only select ~7% of the dataset and remove important signalling events that are represented by phosphopeptides of lower intensities.

2. Their high quality dynamic and quantitative data provided by the authors should be used to perform a network inference analysis via mathematical modeling tools (ODE equations, Bayesian networks...). Given how this methods can be quite time consuming, they could be restricted to a relatively small subset of interesting proteins. This would provide novel biological knowledge of a quality not achieved by the present very superficial computational analyses, and a starting point for future investigations on temperature stresses and on other cell stimulation studies.
We thank reviewer for the suggestion. In the present manuscript, we used simple and accessible tools to correlate dynamic profiles with phosphorylation motif and protein-protein interaction data to establish relationship between kinases and putative substrates. We agree that the comprehensive study of mathematical modeling tools could provide additional benefits and biological knowledge that would not be accessible using our current methods. However, as the reviewer rightly pointed out, the evaluation of these methods are expected to be quite laborious and time consuming, and we believe that a proper analysis of Bayesian networks and other network inference tools would be best addressed in a separate contribution.

3) The authors state that the same processes have been investigated by transcript analysis in the past. These data are not further discussed. It would be interesting to relate the present data to the transcript data or to at least relate the conclusions.
We added the following test along with a supplementary table comparing our phosphoproteomics data with transcriptomics analyses at the beginning of the discussion section to address this point:
Previous transcriptional profiling studies on the heat shock response in yeast identified several differentially regulated genes involved in carbohydrate metabolism, protein folding and degradation, cytoskeletal reorganization, protein synthesis, ribosome synthesis and processing (Gasch et al, 2000; Gasch & Werner-Washburne, 2002). While our quantitative proteomics data indicated that only ~2 % of proteins showed significant changes in protein abundance in the first 30 min upon heat shock, we noted that several proteins including HSP12, HSP26, HSP30, HSP42, HSP78, HSP82, and HSP104 were upregulated by at least 2-fold. We also observed that a subset of ~0.4 % of all quantified proteins including the mitochondrial electron transporter Cyc1 and Cdc25 that regulates RasGTPase activity and cAMP cellular levels were downregulated following heat shock (Table E3). We found that approximately half of the differentially abundant proteins from our proteomics data were also regulated in previous transcriptomics studies (Gasch et al, 2000), and support the activity of important regulatory mechanisms involved in the response to environmental stress. However, the

comparison of genes regulated upon heat shock with dynamic phosphoproteins identified in our study revealed an overlap of only 6% (119/1959), suggesting that phosphorylation dynamics are not necessarily correlated with gene transcription and protein expression (Table E8). These results also underline the significance of protein phosphorylation as a primary response to heat shock.

4) The whole discussion section is weak. For the most part is restates the findings of the results section without achieving a more in depth discussion by relating their data to other datasets and the present knowledge in the field.
The discussion section has been modified to compare our data with those of transcriptomics. We also included a discussion on the significance of Cdc28 tyrosine 19 phosphorylation during the heat shock response to follow-up on our observation based on phosphoproteomics profiles of Cdc28 and its substrates. We believe the discussion has been strengthened from the previous submission. We would like to remind the reviewer that this study is the first describing phosphorylation events following heat or cold stress, and accordingly it is difficult to compare our data with other related studies.

5) The notion that concordance in the temporal profiles is biologically meaningful, in particular for the identification of kinase-substrate relationships is very shaky. The authors remark that themselves on page 10 in the discussion of the clusters. This questions some of the major conclusions and minimally, the basis for the assumption that temporal concordance has functional meaning should be discussed and rationalized. It is a well known fact that kinase substrates have different affinities for kinases and that this is reflected in different kinetic profiles.
This is very important comment. We agree with the reviewer, and kinase-substrate interactions are complicated and difficult to study. We also agree (and also mentioned this on p. 9-10) that kinetics of phosphorylation should be affected by multiple factors. At the same time we are not aware of any in-vivo high resolution studies describing kinetics of phosphorylation. Indeed, the majority of studies are usually reporting in-vitro phosphorylation and changes in protein phosphorylation are often quantified using western blots and often less precise than MS. We make no claim that all substrates of the same kinase should follow exactly the same dynamic profile. We report (quite unexpectedly) that phosphorylation sites on the well known Cdc28 interactors are all displaying similar dephosphorylation profiles upon inhibition of Cdc28 during heat shock. The majority of these phosphorylation sites (9) are well known substrates of Cdc28 but we also detected three new pSites on these proteins that could be therefore putative Cdc28 substrates (Kar9 T598, Msa2 T5 and Ste20 T573, see Figure 4). To further substantiate these finding, we have performed additional experiments where we inhibited Cdc28 in a highly selective fashion by using a CDC28 analog-sensitive endogenously expressed in the same strain background. We performed these experiments using six biological replicates with reverse SILAC labeling and our results confirmed the dephosphorylation of 2 out of 3 putative substrates (Msa2 T5 dephosphorylation (log2)=-4.68, log10(pValue)=-2.48 ; Ste20 T573 dephosphorylation (log2)=-2.38, log10(pValue)=-3.06). Only Kar9 T598 could not be determined due to its low abundance. These new results are now presented in Table E5. We added the following text to p. 11:
To confirm putative Cdc28 substrates we also performed large-scale phosphoproteomics analyses on a yeast strain where the WT Cdc28 was replaced with an analog sensitive mutant Cdc28as expressed in the same strain background (Holt et al, 2009). These experiments were carried out on six biological replicates with reverse SILAC labeling and confirmed the dephosphorylation of 2 out of 3 putative substrates: Msa2 T5 (log_2FC: -4.68, -log_{10}(pValue): 2.48) and Ste20 T573 (log_2FC: -2.38, -log_{10}(pValue): 3.06). Unfortunately, the Kar9 T598 was not detected in these experiments due to its low abundance. The complete list of quantified phosphosites obtained from the inhibition of the analog sensitive mutant Cdc28as is presented in Table E5.

Minor points

Abstract
1. The authors should highlight more biological findings, specifying for instance suggested novel kinase-substrate connections that they could find and that are interesting in the context of temperature shock response. The second sentence contains unnecessary details on transcriptome potential, which is a method they did not use and which is not further discussed.

Discussion relating to transcriptomics data is now presented in the manuscript, and we suggest leaving this sentence for completeness.

Introduction

2. The authors should provide a clear scheme with all the knowledge that is already available from transcriptome profiling studies. In particular, this should include the proteins that will be mentioned in the rest of the manuscript (PKA, Cdc28, Sch9...). Which pathways? Receptors? Kinases? Signaling cascades? Transcription factors? This would greatly ease the understanding of the analyses they perform and the conclusions they draw, which involves many components that participate in several cellular functions.

We do not believe this comparison would be appropriate mainly for two reasons. First, our proteomics data indicated that no kinase or phosphatase displayed significant changes in abundance during the first 30 min of heat/cold stress although they displayed important changes in their phosphorylation. Also, the time frame of our proteomics analyses (30 min) is very different than that of of trascriptomics profile (4 h).

3. First paragraph. They should provide more references. For instance: Aguilera Jaime 2007, Hoffmann Sara 2012, ...

As suggested, we added additional reference where appropriate.

4. Second paragraph. Sentences 2 to 5 are too verbose, and provide information that is only partially relevant to the method they've used. It would be better to shorten it, and give higher focus to the highlights of the paper. At the end of the paragraph they state "We reasoned that the acquisition of high resolution kinetic profiles could provide valuable information not only on phosphoproteome dynamics but also on the correlation of kinase-substrate relationships." As discussed above, the basis for this statement should be minimally rationalized, and given the complex cluster membership also experimentally tested.

We left the sentences on prior studies on cold shock for completeness as they relate to this work. We changed the last sentence to:

We reasoned that the acquisition of high resolution kinetic profiles could provide valuable information on phosphoproteome dynamics to further understand how temporal changes in kinase and phosphatase activities are reflected on downstream substrates in response to environmental stress.

Results

Section "High temporal resolution of the S. cerevisiae phosphoproteome dynamics in response to heat and cold"

5. How many SCX fractions did they use?

We fractionated samples in 7 fractions (including flow through), this is described in Materials and Methods section (under "Phosphopeptide isolation and fractionation" section).

6. Add SILAC protocol reference, either here on in Methods section

An appropriate reference describing the SILAC protocol was included on p.5 of the revised manuscript.

7. Did the author check the temperature before the first sampling?

Yes, temperature was monitored throughout all experiment. Before the first sampling (T0) for all SILAC cultures temperature was 30°C (+/-1°C).

8. Table E1 (file named "62904_0_table_797373_njbk60") should be more self-explanatory. No Sheets should be left without name. What is the content of Sheet1, Sheet2, Sheet3 and Sheet4? What is the meaning of the "Score" columns in the "Phosphopeptides" sheet?

We are sorry for that mistake. All our excel tables contain tab called "description" which contains explanation for all the columns.

9. How did the SILAC scatter plot look like? A few sample scatter plots should be added to supplementary material

We are not sure how this kind of plots can be useful since we have 15 experiments and scatter plot allows us to do only pairwise comparison. We defined dynamic pSites not based on the absolute values of FC but rather on their consistency across experiments. We provide all pairwise

comparisons as a scatter plot matrixes of obtained SILAC ratios below and can include them as a supplementary figure if necessary.

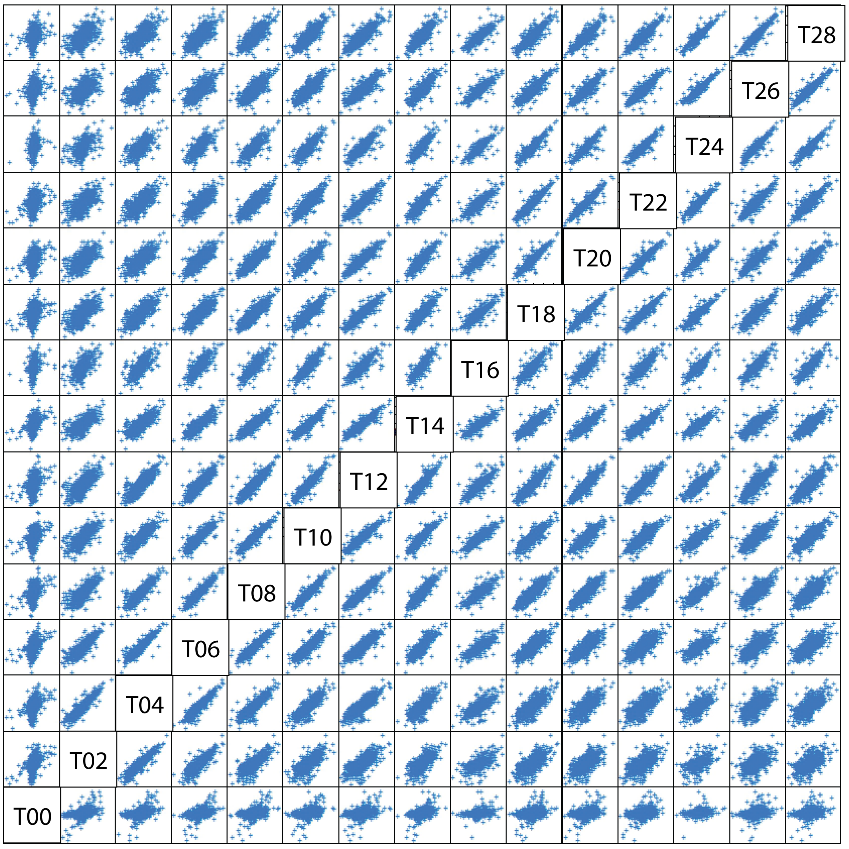

Scatter Plot Matrix

10. The authors should explain how to interpret SILAC fold change ratios for readers that have no experience in the method.
As explained above and on p.7, we don't use SILAC fold change ratios in the selection of regulated phosphorylation sites. Rather, to define biologically regulated phosphosites in the dataset, we first performed fitting of all kinetic profiles with a polynomial model, selecting only those with $R^2 > 0.9$ (Fig 1B and Fig E2). This analysis does not depend on the absolute ratios of abundance measurements or fold change (FC), but it is based on the shape and low variance of kinetic profiles. Therefore, this approach enabled the selection of profiles of low amplitude that could elude conventional quantitative phosphoproteomic methods based on FC alone. Also please see our response to point 14.

11. Please explain why you chose to also plot interquartile ranges, what info they provide.
We needed to determine the global effect of the treatment (heat or cold) on the phosphoproteome. In the case of a single phosphorylation site we could simply use SILAC fold change ratio (FC). In a similar way for a large population of phosphorylation sites we can use width of the corresponding FC distribution that is represented as the interquartile range (IQR). Obviously if the treatment produce changes in phosphorylation it will lead to increase/decrease of FC ratios for phosphorylation sites which in turn will result in the widening of the whole FC distribution. In our case we use IQRs to illustrate progressive large-scale effect of both treatments on the phosphoproteome with time (Figure 2B).

12. Why show the results for non-phosphorylated peptides? The samples were TiO2 enriched, so the data relative to these peptides is not at all meaningful or reliable.
In our case after TiO_2 enrichment we still have ~20% of nonphosphorylated peptides in the sample. We can use data for these nonphosphorylated peptides to determine the global effect of the treatment at the proteome level (see our response to point 11) and compare it with the effect on the phosphoproteome. Comparison (Figure 2B) shows that within our time frame of measurements (30

© European Molecular Biology Organization

min) both heat and cold shock produce much stronger effect on the phosphoproteome compared to protein abundance (which probably would be significantly altered at later time points).

13. (related to previous point) The authors state that "the abundance of non-phosphorylated peptides remained relatively constant over the entire time period of the experiment". This is not relevant and misleading, as one would expect the non-phosphorylated form of a peptide to have an opposite trend with respect to the phosphorylated one.
We are sorry for this misunderstanding. By nonphosphorylated peptides we do not mean the nonphosphorylated counterparts of detected phosphopeptides, it is just peptides that were retained on TiO_2 in a nonspecific manner (see our response to point12).

14. Third paragraph: it's not clear and needs rephrasing. How did you check for LOW VARIANCE? Explain why it can be important to also consider LOW AMPLITUDE profiles.
By low variance we mean low RSD of SILAC measurements that allow us to build kinetic profiles from our data. Those profiles with high RSD will not be fitted well (based on R^2) and therefore are not considered for subsequent analyses. Measured FC value is always a ratio between control and stimulated state, therefore it does not reflect actual stoichiometry of phosphorylation. For example, a FC 10 might reflect change in phosphorylation from 1 to 10% whereas FC 2 might indicate full protein phosphorylation (from 50 to 100%). Therefore there is no reason to believe that profiles with higher amplitude would be more biologically meaningful. Profiles with low amplitude (small FC) could be as important as profiles with high amplitude. In this study we assign significance based on the shape of kinetic profiles rather than absolute values of the corresponding FC which allow us remove amplitude from the equation.

15. Why did you perform the fuzzy clustering here? What's its purpose?
It is a very convenient way to split our kinetic profiles into different types. Also we believe that in our case visualization is more "friendly" compared to hard clustering (e.g. hierarchical clustering) where clusters are typically represented as a trees with the corresponding heatmap. Hard clustering is favourable if clusters are well separated. However, this is not the case for our time-course data, where profile clusters could overlap. Additionally, hard clustering algorithms are often highly sensitive to noise whereas soft clustering is more robust (see: http://www.bioconductor.org/packages/release/bioc/vignettes/Mfuzz/inst/doc/Mfuzz.pdf).

16. Did you set some kind of threshold to distinguish between "static" and "dynamic" profiles? Or how did you do it?
There were several filtering criteria. First we required 10 and more measurements across 15 time points (see our response to point 1 of the major criticism). Then we reasoned that regulated pSites should exhibit consistent temporal trend which should result in better fitting, here we used R^2 threshold of 0.9. Later we performed soft clustering for all profiles with $R^2>0.9$ and kept only those with cluster membership > 0.7. The corresponding phosphorylation sites were defined as "dynamic" and everything else was considered "static". Like any other type of data analysis it does not provide us with 100% confidence (e.g. there could be regulated pSites that ended up in "static" category because they did not pass the filtering) but we tried to do our best to define subset of regulated pSites.

17. Figure E4 (end of third paragraph) is called before Figure E3.
We changed the order so that Figures E3 and E4 are called sequentially.

18. Please comment on the results you obtain, with a focus on general and temperature stress responses - the GO categories (can you provide some sample proteins from the main categories, and comment on them?), the kinases and phosphatases you find to be regulated (they are not too many).
We now provide uniprot accession numbers for all dynamic phosphoproteins overrepresented in for different GO categories in Table E9.

Section "Temporal phosphorylation profiles identify potential kinase substrates in response to heat shock and cold stress"

19. This section is very long and it includes several different analyses. It would be much better to divide it into smaller sections, each one with a clear and progressive focus.

© European Molecular Biology Organization

This section is only 2 pages longer than the previous one, and we did not deem necessary to break it down more to avoid losing the focus of this section.

20. In line 6 it talks about FC, while at line 7 it says IQR. Are they both correct? From my understanding of the sentence, IQR should be FC.
On p. 9 of the revised manuscript, his sentence was changed to:
…the temporal variation in FC (increase or decrease) of several profiles reached a plateau within 20 min upon temperature shift, consistent with global changes observed previously on IQR measurements (Fig 2B).

21. Tsl1 and TSP should be mentioned in the introduction, as their involvement in heat response was previously known. Your result, which supports the reliability of your data, would not be easily registered and remembered.
We include the following sentence in the introduction and made appropriate reference to previous work on Tsl1 and TSP complex on p.9.
Exposure to low temperature induces the accumulation of trehalose, glycerol, and heat shock proteins to protect yeast cells against freeze injury (Schade et al, 2004).

22. Second paragraph. This paragraph needs to be restructured. First observe that in the case of Cdc28 Y19 (inhibition phospho-site) you noticed how its known targets have a specular behavior. Then suggest that, by looking at phospho-sites that cluster with together under the same putative kinase group, you may therefore be able to find novel targets of certain kinases. I would therefore start with the Cdc28 example, as it is more logical. The fact that phospho-sites of important kinases cluster in different groups doesn't necessarily point in the same direction.
In the 2nd para. it is important to first discuss the global changes on kinases and phosphatases before presenting specific data on Cdc28. We argue that it may not be appropriate to enter the discussion of these data by focussing on Cdc28 without discussing global changes on these enzymes.
23. Second paragraph. Why only the downstream proteins?
Here we speak of the substrates of kinases and phosphatases. There are no upstream substrates. We removed the word downstream to avoid ambiguity.

24. Second paragraph. Please, better explain the meaning of a "putative kinase group".
This follows from KinomeXplorer results. Basically it is kinase group associated by KinomeXplorer with default filtering criteria, therefore we replaced "putative" by "assigned".

25. Second paragraph. Please, provide a (supplementary at least) table with (indicatively) the following columns: phospho-site, protein, putative kinase group(s).
This information is presented in Table E1 (column "NetworKIN Kinase Group(s)").

26. Second paragraph. The section that starts with "Next, we regrouped dynamic phosphosites associated to specific kinases..." needs a clearer explanation. If I got it right, it says that you checked if the phospho-sites that were found to be phosphorylated by the same putative kinases were grouped in the same cluster (had the same dynamic). Is this right?
This is correct. We changed the corresponding sentence to:
Next, we regrouped dynamic phosphosites associated to specific kinases with their corresponding clusters to determine if phosphosites that were found to be phosphorylated by the same putative kinases were grouped in the same cluster.

26. Second paragraph. "... we also noted that several dynamic sites were predominantly observed in clusters of selected kinase groups" is not of clear interpretation. Do you mean to say that some clusters/dynamics were more frequently represented, in several kinase groups?
This sentence was changed to:
While temporal profiles associated to specific kinases appeared to be complex, we also noted that several dynamic sites/clusters were more frequently represented in selected kinase groups (Fig 3C).

27. Second paragraph. What happens if you group together the specular clusters (the ones that are already paired in Figure 5)? They could be treated as the same, in a way, as a dephosphorylation may induce an equivalent phosphorylation, for instance, if the dephosphorylation activated a kinase.

During initial data analysis we tried to use different number of clusters and group similar clusters together which didn't improve our results.

28. Third paragraph. This somehow belongs to the previous analysis of Cdc28, which should come before the rest.
See response to point 22 above.

29. Third paragraph. Can you comment on the results you obtain? What can you conclude from these results?
We are not clear on which results the reviewer wants us to expand and comment upon.

30. Fourth paragraph. Here the authors compared two very different datasets. What is the aim of this comparison?
The comparison with Bodenmiller's data was requested in a preliminary evaluation of this manuscript and provides an independent comparison of different large-scale datasets where changes in kinase activities are observed.

31. Fifth paragraph. Msn2/4 were both already associated with heat shock, you should underline that.
This is highlighted in the following sentence on p.12:
Among these are known examples of transient dephosphorylation of Msn2 and Msn4, two transcription factors that are activated upon cellular stress conditions, and rapidly undergo nuclear translocation (Jacquet et al, 2003).

32. Fifth paragraph. Please comment on the model shown in Figure 5. What is new about it?
It is not a detailed model; here we just attempted to overlay known literature information with our data. We could observe that set of functionally related proteins also exhibit similar phosphorylation dynamics which again is rather unexpected (see our response to point 3 of the "Major points").

33. Figure 5. Can you comment on the fact that most of the phospho-sites shown here are quickly and transiently DOWN-regulated by heat, while they are only mildly UP-regulated by cold? It's a very interesting and consistent behavior in response to both temperature stresses. And did you try to look at which phospho-sites cluster with Msn2/4?
Indeed we observe very different behaviour for heat and cold stress. It is interesting but we do not know how to rationalize this observation. However, we commented on these observations at the end of this section (p.16) and in the discussion (p.20). Msn2/4 profiles belong to cluster number 1, all of its members can be found in Table E2 using column "Cluster_ID", the large majority of them are presented on Figure 5.

34. Sixth paragraph. In this and other sections, the authors hypothesize a few novel interactions, which appear here and, sometimes, later in the text as they proceed with their investigation. This leads to confusion. Some of these could be used to generate a model involving several components, which could be used to suggest novel signaling processing mechanisms. You can try to provide a few more schemes like the one in Figure 5, at least those that you believe to be the most relevant.
Due to the lack of similar studies (signaling event triggered by temperature stress) this exercise could only be speculative. In order to present meaningful models we would have to go much deeper into functional validation through mutagenesis and biochemical assays which is outside of the scope of the present manuscript.

35. Sixth paragraph. Can you comment on the fact that several glucose and glycogen metabolism enzymes are involved in the response? Was it expected or is it a surprise?
This is commented upon in the following sentences:
Rgt1 regulates the expression of HXT genes encoding glucose transporters, and is phosphorylated *in vitro* by all three isoforms of PKA at serine residues located within consensus sites (Kim & Johnston, 2006). Igd1 is also involved in glucose metabolism and was reported to act as an inhibitor of Gdb1p by enhancing the ability of yeast cells to store glucose as glycogen. The phosphorylation of Igd1 could regulate its interactions with Gdb1 (Walkey et al, 2011).

Section "Cross-talk of protein phosphorylation between heat shock and cold stress"

© European Molecular Biology Organization

36. The conclusion to the first paragraph (the last sentence) is somewhat obvious. Can you speculate on its role during response to heat/cold shock?
This sentence reflects the observation made from our phosphoproteomics data:
Our data suggest that phosphorylation of Nth1 might regulate its enzymatic activity to control intracellular levels of trehalose.
Unfortunately, we do not have any structural information on Nth1 to determine how changes in phosphorylation might affect its activity. We feel that going beyond this observation might be highly speculative and not appropriate for this results section.

37. Check the percentage you give in the second paragraph. Shouldn't it be 16% too be consistent with the other percentage you give in the rest of this section?
We change the number accordingly on p. 14 of the revised manuscript.

38. Did you also detect Hsp12? Hsp60? Hsp1? If so, what about them?
We haven't detected any phosphosites on Hsp12/60/1.

39. "Cdc28 is inhibited under heat shock and cold stress..." Can you give a reference to this fact?
An appropriate reference was added to this sentence on p. 10
40. This section does not really discuss cross talk.
We changed the title of this section as follow to avoid confusion relating to cross talk:
Differential regulation of signaling pathways upon heat shock and cold stress

Discussion
40. You should highlight the importance of looking at the shape of a dynamic curve, and mention which information can be derived from it.
The comment on the analysis of the shape of the dynamic curve is captured in the following sentence on p.19):
The ability to monitor changes in protein phosphorylation with high temporal resolution facilitated the identification of regulated phosphosites based on the shape of kinetic profiles rather than conventional approaches that typically rely on FC measurements. This provided an inherent advantage in the identification of sites that displayed small but tractable changes in phosphorylation.

41. Can you comment on the choice of using a 2 minutes time resolution? How much would it be possible to reduce it in order to perform smaller experiments that still provide a similar amount of information - that is, without compromising the dynamic shapes you estimated from your measures?
Temporal resolution should be chosen in a way that minimizes chances of having any maxima/minima between measurements. Recently we completed similar dynamic study with 5 sec resolution (with much shorter time frame of only 60 sec) which revealed only monotonous profiles (Kanshin et al. Cell reports, 10, 1202-14, 2015). From this we concluded that we need at least several minutes for phosphorylation to go up and down and 2 min resolution appeared optimal.

42. Once again, do not only say that your study showed that by clustering the dynamic profiles you can hypothesize new interactions, but also and especially that with the data you have collected you could provide an advance in the knowledge on heat/cold shock response and on signaling integration mechanisms.
The following sentence was added on p.19 of the discussion:
The clustering of kinetic profiles enabled the grouping of new potential interactions between kinases and putative substrates, to further understand the integration of signaling pathways and the response to environmental stress.

43. Can you comment on the fact that a much larger number of phospho-sites is responding to heat rather than cold shock? Why do you think that happened? Was the temperature not low enough?
This is commented upon on p. 21:
Cell response to heat shock and cold stress was also reflected by an uneven distribution of dynamic phosphosites largely favoring the former stimulus. The observation that approximately 20 % of all dynamic phosphosites were observed upon cold stress is consistent with lower enzymatic activities and metabolic rates expected in yeast at temperatures below optimal growth conditions.

Materials and Methods
44. Which protein:trypsin ratio did you use?
We used 1:50 (w/w) ratio, now we added this to Material and Methods section p. 24.

45. Phosphopeptide isolation protocol. Which buffers did you use for equilibration? And for elution?
We added the following description on p.24:
We equilibrate TiO2 material in 250 mM lactic acid in 70% ACN 3% TFA, the same buffer was used for sample loading. Phosphopeptides were displaced from TiO_2 with 500 mM phosphate buffer at pH=7. Complete protocol can be found in a recent publication from our group (Kanshin et al, 2013).

Figures
46. Figure 2B. In the y-axis, is that IQR or log2FC? In the caption it talks about FC
It is IQR – a measure of how wide is the corresponding FC distribution. The caption was corrected accordingly.

47. Figure 2C. What's the meaning of the "node degree"?
Node degree is the number of interacting partners in the network and is now mentioned in the figure caption.

48. Figure 5. Sch_S726 is missing a "9" (Sch9).
Change made on the figure.

49. Figure 5. What's the meaning of the dotted connections? Which of these links were made or supported by your data?
The dotted connections are based on previous protein-protein interactions and from our observations suggesting that decrease PKA phosphorylation could inhibit transport through nuclear pore complex (NUP) and Skn7 activity.
50. Figure E3. What's -Ig[PValue(Benjamini)}?
It is negative log10 of pValue corrected for multiple hypothesis testing using Benjamini criteria. Change made on the corresponding Figure legend.

51. Figure E6. It's not easy to understand which cluster the top-row bars correspond to. Also, what do you mean by "distribution of substrates"?

We updated the figure to make it more readable.

Reviewer #3:

The present study revolves around the use of SILAC based phosphoproteomics to unravel the molecular responses to heat- and cold stress in the yeast Saccharomyces cerevisiae. The authors cultured an arginine and lysine auxothrophic yeast strain labeled with various stable isotopes of arginine and lysine in order to quantify relative differences in peptide levels after heat- or cold shock. Thus, at time point 0 the authors add media of different temperatures to induce temperature changes in the cultures and every second minute they harvest cells to examine the phosphoproteome by mass spectrometry after enrichment of phosphopeptides by TiO2 chromatography.

The study is technically very well executed and the strength of this study is clearly the large number of time points, which allow the authors to perform very thorough bioinformatics analyses. This analysis identify among others the cell cycle kinase Cdc28 and nutrient sensitive signaling kinases including PKA, TORC and Sch9 signaling as well as stress-regulated transcription factors Msn2/Msn4 as important regulatory circuits involved in the response to heat and cold stress.

Although this is a very interesting there are a number of issues that must be addressed.

Major points:
To induce the temperature shifts the authors add a large volume of cold or hot media, while the control media receives 30 degrees media. Although similar dilutions are induced of the cultures, it does not appear at what growth stage and to what density the cultures changes after dilution. Are the

cells exponentially growing? Details in how cell culturing was carried out should be provided. Nonetheless, the volume added is larger than the volume of each culture resulting in at least halving the cell density. This means that the growth rate of the cells also change, and it must be expected that cells that are either cold or heat shocked do not grow, only the control cells will grow. This will inadvertently affect the conclusions that can be drawn from this study as the interpretation of the temperature change may be due to effects of growth. Therefore, the authors should show that the observed changes are caused by a change in temperature and not simply caused by a changed growth
rate.
Our cultures were treated at OD_{600}=0.8 and they were diluted with fresh media approximately~1.66 times. Typically @ 30°C in SD media cells divide every ~ 2h and we could expect only about 18% increase in the number of cells in our control culture (if heat and cold shock stop cell proliferation immediately) and only in the last time point (taken @ 28 min). This is a relatively small deviation and its effect disappears during data analysis since all the isotopic ratios are normalized according to total protein amount per SILAC channel. This is a standard step of data processing in MaxQuant, this normalization is done separately for every time point. Unfortunately we do not see any other way to perform this experiment in a better way – we absolutely need unstressed control even if cells will grow faster compared to heat/cold conditions, relatively short time frame is an advantage in this case.

Moreover, as the main conclusions of the study seem to relate to Cdc28 functions and signaling related to nutrient sensitive pathways the effects of changes in temperature may just reflect a consequence of altered nutrient uptake, which affects cell cycle and hence Cdc28 activity, and hence not be an affect induced by temperature changes per se. Thus, how does the temperature changes affect nutrient uptake and metabolic fluxes e.g. incorporation of nutrients in to storage molecules and/or degradation of nutrients.
It is unlikely that we increase nutrient uptake by diluting our media because for dilution we use exactly the same media (same batch) so the concentrations of all the nutrients remain the same. Also our cultures are not in stationary phase and dilution should not result in changes in growth rate. If we consider Cdc28 example we could explain its inhibition by decrease in nutrient uptake upon heat and cold stress, but the primary reason should be stress associated with change in growth conditions (temperature).
At the same time we are trying to address issue of stimulus-specific vs. stimulus-independent signaling events by analysing kinetic profiles (see Figure 4A). For example if we see the same effect upon both heat and cold stress we hypothesize that phosphorylation is associated with the general stress and does not depend on the direction of the temperature shift ("temperature-independent" group on Figure4A).

It does not appear if phosphopeptides have been normalized to the level of the total level of the same protein. Although it is the immediate response that has been examined, it cannot be excluded that protein levels are affected even after a few minutes. The authors should explain how each phosphorylated protein has been normalized.
On the figure 2B we are using nonphosphorylated peptides (nonspecific binding on TiO_2, ~20% from all ids) detected in our study to show that both heat and cold stress induce larger changes on the prosphoproteome compared to proteome. Whereas this is valid on a large scale, reviewer is correct that some of the proteins might change their abundance significantly even within relatively short time frame of our experiment. Therefore we must normalize our pSite quantification by the protein abundance. We kept flow-through fractions containing nonphosphorylated peptides from TiO_2 enrichment experiments, desalted them and performed offline HPLC fractionation on PGC (Hypercarb™ Porous Graphitic Carbon) column collecting 40 fractions that were subsequently analyzed by MS (results are presented in Table E3).This allowed us to quantify ~ 85 % from all our dynamic phosphoproteins (reported in Table E2). We would like to mention that only for 2 of them we observed >2 fold changes on a protein level. Therefore HSP42 (log2(FC)=2.06) and ZEO1 (log2(FC)=1.33) were excluded from the list of dynamic phosphoproteins.

Finally, the authors discuss the identified pathways and specific signaling components well in accordance with the literature although a few recent studies using SILAC based proteomics to examine starvation responses in yeast seem to have been omitted. However, none of their findings have been functionally validated, which certainly would increase the interest of the work. I do not

expect the authors to examine the function of specific phosphorylation sites, but heat/cold shock responses should be examined in selected mutants carrying defects in relevant signaling pathways.
We performed follow up validation for Cdc28 substrates. Based on kinetic profiles we suggested 3 novel putative substrate pSites of Cdc28 (Figure 4A). Now we completed experiment where Cdc28 was selectively inhibited by using an analog sensitive form of the protein. We were able to detect and quantify 2 of these putative substrates. Our results show strong dephosphorylation of both sites (Msa2 T5 log2(FC)=-4.68, log10(pValue)=-2.48 / Ste20 T573 log2(FC)=2.38, log10(pValue)=-3.06) upon Cdc28 inhibition which is another confirmation that they could be direct Cdc28 substrates. Kar9 T598 unfortunately was not detected. Complete results of these experiments are now presented in Table E6.
As discussed previously, we also complemented these studies with functional assays on a yeast strain where the WT Cdc28 was replaced by a cdc28-af mutant defective for Y19 and T18 phosphorylation and determined its cell cycle profile after heat shock. These results indicated that the increased phosphorylation of Cdc28 Y19 and associated reduction in kinase activity observed during the heat shock response restrain cell cycle progression, most likely to allow time for cells to adapt to the cellular stress associated with increased temperatures and to promote effective cell division. These new data are now part of a separate section on p. 18 of the revised manuscript.

Minor points:
The authors should show in a suppl figure, which motif sequences that have been enriched in their analyses.
We were not able to perform this analysis due to low number of dynamic pSites. Particularly it would be extremely helpful to see if there are different sequence motifs enriched in different clusters (dynamic behaviours). Unfortunately like with any other enrichment analysis results are greatly affected by the population size, therefore we were not able to get good confidence values due to very small number of pSites in each dynamic cluster (10-60 pSites).

2nd Editorial Decision 22 April 2015

Thank you again for submitting your revised work to Molecular Systems Biology. We have now heard back from the two referees who accepted to evaluate the study. As you will see, the referees are now globally supportive. They still raise however a series of points that we would ask you to carefully address in a revision of the present work. The recommendations provided by the reviewers are very clear and there is no need for further experimentation.

Reviewer #1:

The authors have to a large degree accomodated the issues that I addressed initially. However, studying shock responses (to heat and cold), it also implies that the responses can return to the starting point prior to induction of the shock. Therefore, has the authors examined how many of the phosphorylation changes that can return to the starting point if they reverse the temperature change? This would be an interesting add on to an otherwise well conducted study.

Reviewer #2:

Review of revised version of MSB-15-6170, "Phosphoproteome dynamics of Saccharomyces cerevisiae under heat shock and cold stress" by Thibault and colleagues.

In this revised version, the authors responded satisfactorily to some of the concerns raised in the original version. There are still some issues that would be important to resolve.

Main concerns:
1. Tab_E1. Heat.T16 and Heat.T24 have: 62% and 55% NA, and the same percentage for Cold.T16 and Cold.T24. Why are the percentages for heat and cold exactly the same? Is it a problem relative to the light-SILAC-reference? And, most importantly, what is the reason behind such a high number of NA? Was there a problem during the MS measurements? Please, clarify this issue and, if the MS measurement didn't run properly for some reason, consider discarding these data from your analyses.
2. One of the concerns was - and still is - related to the way the study is explained in the manuscript.

In certain cases, which were highlighted in the previous round of revision, the structure of the manuscript renders the content quite difficult to grasp and understand. The authors chose not to perform any improvement in response to this criticism. The authors are encouraged to consider that the message they are trying to convey to a scientist-reader may be lost, unless an attempt is made to present the text more clearly and logically. The paragraph that, by far, is in greatest need of restructuring is the one entitled "Temporal phosphorylation profiles identify potential kinase substrates in response to heat shock and cold stress".
3. While the answers provided to the questions raised are generally satisfactory and complete, the aim of these questions was mainly to highlight unclear points within the paper. The authors are requested to make the pertinent changes also in the manuscript in order to clarify the points that are not clear to the reviewer also to the reader. In particular, the manuscript would benefit from a few explanatory sentences relative to the following questions of the first round of reviews:
Question 5. How many SCX fractions... This is not obvious from the M&M section.
Question 7. Did the author check the temperature...
Question 11. Please explain why you chose to also plot interquartile ranges...
Question 13. (related to previous point) The authors state that "the abundance...
Question 14. Third paragraph: it's not clear...
Question 24. Second paragraph. Please, better...
Question 41. Can you comment on the choice... The response to this question, in particular, is very interesting indeed. It would be very nice to add it to the manuscript.

4. Previously it was suggested to extend the Introduction by adding information regarding previous knowledge on yeast response to heat and cold, with a special reference to the proteins that are discussed later in the manuscript, in order to help the reader understand the biology behind the data. The Authors' reply was that no one has done a phospho-proteomics study on this topic before, and that published studies were not relative to their same time-frame. These are not good reasons not to provide a SHORT background on the biological system being studied.
5. The authors were asked to provide "a few" SILAC scatter plots, not all of them, at least not for the main text (rather maybe as supplemental information). The figure they enclose in their point-by-point response is rather useless in its present form. 2-3 significant examples will be more than enough. The purpose of this is to provide the reader with a simple tool to evaluate the quality of the SILAC experiments.
6. Table E9 does not contain GO categories info (as the authors stated in response to my question 18), at least not in the version that was submitted.

Revision received 1 May 2015

Response to review of Manuscript "Phosphoproteome dynamics of *Saccharomyces cerevisiae* under heat shock and cold stress"
Italic highlights response on specific comments raised by the reviewers
***Bold** type indicates changes to the manuscript, also highlighted in yellow on the revised manuscript*

We thank both reviewers for their constructive comments and positive feedback on our work. We addressed their comments as described below.

Reviewer #1:

The authors have to a large degree accommodated the issues that I addressed initially. However, studying shock responses (to heat and cold), it also implies that the responses can return to the starting point prior to induction of the shock. Therefore, has the authors examined how many of the phosphorylation changes that can return to the starting point if they reverse the temperature change? This would be an interesting add on to an otherwise well conducted study.
Indeed this would be an interesting experiment to see the reversibility of phosphorylation changes upon "pulse" stimulation with the temperature. Obviously, additional experiments would be required to differentiate adaptation to temperature with those associated with a plasticity of the response once temperature is reversed. This would require repeating the whole study using new experimental setup (heat shock->measurement->return to 30C->measurement), and would be best addressed in a separate study. From our current dataset, we determined that a number of clusters

showed an adaptation like response where changes in phosphorylation returned to the starting point under increase or decrease in temperature. Examples of this are presented in Figure 3A and dynamic profiles on Figure 5. We also added the following text on p. 10 of the revised manuscript:
Several profiles showed changes in phosphorylation that returned to their original status within 30 min post stimulation. For example, this is the case for the kinase Hog1, and the transcriptional factors Msn2 and Msn4 that were previously reported to be regulated by changes in temperature (Panadero et al, 2006).

Reviewer #2:

In this revised version, the authors responded satisfactorily to some of the concerns raised in the original version. There are still some issues that would be important to resolve.

Main concerns:
1. Tab_E1. Heat.T16 and Heat.T24 have: 62% and 55% NA, and the same percentage for Cold.T16 and Cold.T24. Why are the percentages for heat and cold exactly the same? Is it a problem relative to the light-SILAC-reference? And, most importantly, what is the reason behind such a high number of NA? Was there a problem during the MS measurements? Please, clarify this issue and, if the MS measurement didn't run properly for some reason, consider discarding these data from your analyses.
We thank the reviewer for the opportunity to provide further details on our dataset. With very few exceptions, the proportion of NAs in heat and cold should be equal. The reason for this is accounted for by our experimental design, SILAC quantification and MaxQuant data process. Our metabolic labeling experiments used 3 SILAC channels for control (Light), cold (Medium) and heat (Heavy) shock. For every time point cell cultures were pooled together in excess of cold ethanol. During the MS analysis each peptide will be represented as a triplet in the MS-scan and a ratio of intensity between Light, Medium and Heavy forms will be used for quantitation. We also use the function requantitate in MaxQuant to integrate signal at expected m/z and time coordinates for peptide clusters (within the triplet) that were not observed (e.g. intensity is lower than a background). Accordingly, if the whole triplet is not observed or not properly resolved (e.g. due to low intensity of the signal or overlapping signals from other peptides) isotopic ratios cannot be calculated – so both Heavy/Light (Heat shock) and Medium/Light (Cold stress) will not be measured.
We examined the number of quantified phosphopeptides across all time points and determined that T16 and T24 had approximately 50% lower quantified phosphopeptides compared to other time points as shown in the following figure.

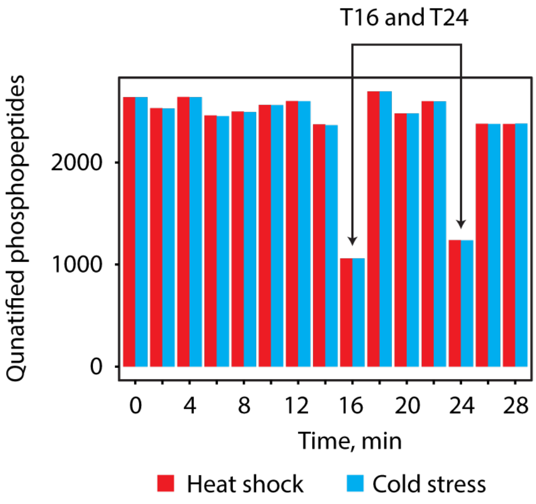

The reason for this lower number of quantified phosphopeptides is unstable spray conditions experienced during overnight LC-MS/MS runs of one SCX salt fraction for each of the corresponding time point (25 mM in T16 and 15 mM in T24). To avoid using compromised data, the LC-MS/MS data for these time points were NOT used in our quantitative analyses. It is noteworthy that salt fractions 15 mM and 25 mM typically have a higher number of phosphopeptides compared to other fractions and the overall impact on the number of quantifiable phosphopeptides per time point is more important for these fractions compared to others. For example, we also experienced

unstable spray for salt fraction 80 mM from T8, but the impact on quantifiable peptides was barely noticeable (see Figure below).

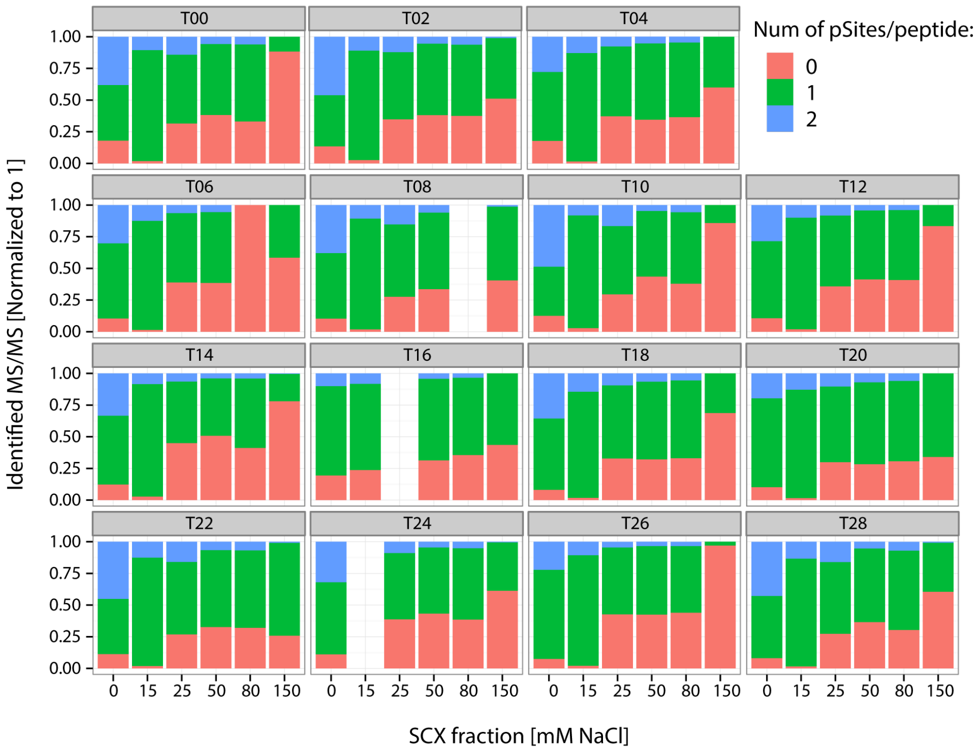

To evaluate the impact of these missing fractions on the number of phosphopeptide profiles, we imputed randomized data based on an average number of NAs from other time points for fraction 25 mM in T16 and fraction 15 mM in T24, and determined how many profiles (with RankN>9) would have been lost.
Based on this model, we estimated that a total of 2932 phosphosites would have been obtained if we could have used these two missing fractions. This represents a 5.6% increase over the 2777 phosphosites that we obtained in our current dataset. While a slight increase in phosphopeptide coverage is anticipated from these missing fractions, we do not expect that these additional data would change the conclusions and outcome from our bioinformatics analyses (e.g. PPI, GO, kinase-substrate relationships).

2. One of the concerns was - and still is - related to the way the study is explained in the manuscript. In certain cases, which were highlighted in the previous round of revision, the structure of the manuscript renders the content quite difficult to grasp and understand. The authors chose not to perform any improvement in response to this criticism. The authors are encouraged to consider that the message they are trying to convey to a scientist-reader may be lost, unless an attempt is made to present the text more clearly and logically. The paragraph that, by far, is in greatest need of restructuring is the one entitled "Temporal phosphorylation profiles identify potential kinase substrates in response to heat shock and cold stress".
We attempted to address major concerns expressed by this reviewer in the 1st round of revision. We made modification to the paragraph identified by this reviewer and change the text at the bottom of p.10 as follow:
To gain further insights on kinetic profiles shared among dynamic phosphosites, we analyzed our dataset using fuzzy c-means clustering, and selected only clusters with membership >0.7 (Fig 1B, and Fig 3A). These analyses enabled the separation of 388 dynamic phosphosites from 271 proteins into 15 different clusters with unimodal distributions showing monotonous (clusters 5, 6 and 9-12) and adaptation-like (clusters 1-4, 7, 8 and 13, 14) profiles (Table S2). With the exception of clusters 9-12, the temporal variation in FC (increase or decrease) of several profiles reached a maximum value within 20 min upon temperature shift. Several profiles showed changes in phosphorylation that returned to their original status within 30 min post stimulation. For example, this is the case for the kinase Hog1, and the transcriptional

factors Msn2 and Msn4 that were previously reported to be regulated by changes in temperature (Panadero et al, 2006).

3. While the answers provided to the questions raised are generally satisfactory and complete, the aim of these questions was mainly to highlight unclear points within the paper. The authors are requested to make the pertinent changes also in the manuscript in order to clarify the points that are not clear to the reviewer also to the reader. In particular, the manuscript would benefit from a few explanatory sentences relative to the following questions of the first round of reviews.
We are most grateful for the suggestion of the reviewer, and incorporated our response in the revised manuscript for the following points:

Question 5. How many SCX fractions...
We did fractionate our sample in total of 7 SCX fractions, but the last salt fractions of 1000 mM NaCl did not contain any peptides and therefore we haven't include them into analysis.The following text was added to Materials and Methods under the section "Phosphopeptide isolation and fractionation".
Columns were washed with 50 µl of loading buffer and peptides were eluted in 6 separate 100 µl salt steps of 0, 15, 25, 50, 80 and 150 mM NaCl. All fractions (including flow-through) were collected, dried in a speedvac, resuspended in 15 µl of 4% FA prior to MS analysis.
The following text was added to ***p.7 of the revised manuscript:***
and fractionated offline into 6 SCX salt fractions prior to LC-MS/MS analysis
Question 7. Did the author check the temperature...
The following text was added to Materials and Methods under the section "Cell culture".
The temperature was monitored throughout all experiments. The temperature of all SILAC cultures was 30°C (+/-1°C) prior to heat or cold shock.
Question 11. Please explain why you chose to also plot interquartile ranges...
The following text was included on p. 8 of the revised manuscript.
Global effects of the treatment (heat or cold) on the phosphoproteome was evaluated from the width of the log_2 FC distribution of all phosphopeptides for a given time point. The width of this distribution is represented as the interquartile range (IQR). If the treatment produces global changes in phosphorylation this will be reflected by a widening of the FC distribution. We plotted the interquartile ranges (IQR) for log_2(FC) distributions of phosphopeptides to monitor the global changes of the phosphoproteome upon heat and cold stress (Fig 2B). The IQR values of nonphosphorylated peptides retained on TiO_2 in a nonspecific manner are also shown for comparison.

Question 13. (related to previous point) The authors state that "the abundance...
See point above.

Question 14. 14. Third paragraph: it's not clear and needs rephrasing. How did you check for LOW VARIANCE? Explain why it can be important to also consider LOW AMPLITUDE profiles.
The following text was included on p. 8-9 of the revised manuscript.
This analysis does not depend on the absolute ratios of abundance measurements or fold change (FC), rather it is based on the shape and low variance (i.e. low RSD values of SILAC measurements) of kinetic profiles. Profiles with high RSD do not generally provide meaningful R^2 values, and are not considered for subsequent analyses. It is noteworthy that FC values do not reflect actual stoichiometry of phosphorylation. For example, a FC value of 10 might indicate a change in phosphorylation from 1 to 10% whereas a FC value of 2 could correspond to full protein phosphorylation (from 50 to 100%). Since biological relevance of phosphosite cannot be based on FC measurements alone, profiles with low amplitude (small FC) could be as important as profiles with high amplitude (high FC). In this study, we assign significance based on the shape of kinetic profiles rather than absolute values of the corresponding FC values.

Question 24. Second paragraph. Please, better...
The following text was included on p. 11 of the revised manuscript:
To test this proposal, we first associated dynamic and static phosphosites with assigned kinase groups based on predictions made by KinomeXplorer (Horn et al, 2014), a platform that integrates an improved NetworKIN algorithm for modeling kinase signaling networks by combining linear motif analysis with protein-protein interactions.

© European Molecular Biology Organization

Question 41. Can you comment on the choice...
The following text was included in the Discussion section on p. 20 of the revised manuscript:
A temporal resolution of 2 min was selected to minimize the occurrence of minima/maxima between measurements. In a recent study on osmotic shock response in yeast, we monitored changes in protein phosphorylation over a one minute period with 5 sec resolution, and identified only monotonous profiles (Kanshin et al, 2015). Changes in phosphorylation including those associated with adaptation response likely take place over several minutes, and 2 min resolution was deemed optimal to detect these changes.

4. Previously it was suggested to extend the Introduction by adding information regarding previous knowledge on yeast response to heat and cold, with a special reference to the proteins that are discussed later in the manuscript, in order to help the reader understand the biology behind the data. The Authors' reply was that no one has done a phospho-proteomics study on this topic before, and that published studies were not relative to their same time-frame. These are not good reasons not to provide a SHORT background on the biological system being studied.
We include the following text in the introduction on p. 3 to account for changes in kinase activity upon heat shock response.
Short-term exposure to moderate heat shock has been reported to induce a complx cellular response culminating in a transient cell cycle arrest at G1/S transition in both yeast and mammalian cells (Kuhl & Rensing, 2000). This response appears to be associated with the activity threshold of cyclin-dependent kinases (Kuhl & Rensing, 2000), and overexpression of G1 cyclins in yeast has been shown to overcome the transient arrest induced by heat shock (Rowley et al, 1993). Moreover, Ste20, Cla4, and Skm1, all members of the p21-activated kinase (PAK) family, are also activated upon changes in temperature, and play important roles in the regulation of osmosensing, filamentous growth and septin ring assembly (Gancedo, 2001; Holly & Blumer, 1999; Raitt et al, 2000b; Versele & Thorner, 2004). Interestingly, decreased levels of the molecular chaperone Hsp90 or its kinase-specific chaperone Cdc37 are known to affect Cdc28 and Cla4 stability (Farrell & Morgan, 2000), a condition that favors a subset of cells to switch to a filamentous form in response to hostile environment (Hsieh et al, 2013).
We include Tsl1 and TSP in the introduction on p. 4, as their involvement in heat response was previously known.
Exposure to low temperature induces the accumulation of trehalose, glycerol, and heat shock proteins to protect yeast cells against freeze injury (Schade et al, 2004).

5. The authors were asked to provide "a few" SILAC scatter plots, not all of them, at least not for the main text (rather maybe as supplemental information). The figure they enclose in their point-by-point response is rather useless in its present form. 2-3 significant examples will be more than enough. The purpose of this is to provide the reader with a simple tool to evaluate the quality of the SILAC experiments.
We tried to avoid any subjectivity if we were to arbitrary select specific time points for comparison. Supplementary material now include Figure S2 that shows pairwise comparison of SILAC ratios across all time points. We also added R^2 values to the corresponding plots. As expected correlation between quantitative measurements is better for close time points compare to distant ones.

6. Table E9 does not contain GO categories info (as the authors stated in response to my question 18), at least not in the version that was submitted.

GO information is now include in Table S4.

Acceptance letter 18 May 2015

Thank you again for sending us your revised manuscript. We are now satisfied with the modifications made and I am pleased to inform you that your paper has been accepted for publication.
